# Neuroarchitecture of Peptidergic Systems in the Larval Ventral Ganglion of *Drosophila melanogaster*

**DOI:** 10.1371/journal.pone.0000695

**Published:** 2007-08-01

**Authors:** Jonathan G. Santos, Matthias Vömel, Rafael Struck, Uwe Homberg, Dick R. Nässel, Christian Wegener

**Affiliations:** 1 Emmy Noether Neuropeptide Group, Animal Physiology, Department of Biology, Philipps-University, Marburg, Germany; 2 Animal Physiology, Department of Biology, Philipps-University, Marburg, Germany; 3 Department of Zoology, Stockholm University, Stockholm, Sweden; University of Arizona, United States of America

## Abstract

Recent studies on *Drosophila melanogaster* and other insects have revealed important insights into the functions and evolution of neuropeptide signaling. In contrast, in- and output connections of insect peptidergic circuits are largely unexplored. Existing morphological descriptions typically do not determine the exact spatial location of peptidergic axonal pathways and arborizations within the neuropil, and do not identify peptidergic in- and output compartments. Such information is however fundamental to screen for possible peptidergic network connections, a prerequisite to understand how the CNS controls the activity of peptidergic neurons at the synaptic level. We provide a precise 3D morphological description of peptidergic neurons in the thoracic and abdominal neuromeres of the *Drosophila* larva based on fasciclin-2 (Fas2) immunopositive tracts as landmarks. Comparing the Fas2 “coordinates” of projections of sensory or other neurons with those of peptidergic neurons, it is possible to identify candidate in- and output connections of specific peptidergic systems. These connections can subsequently be more rigorously tested. By immunolabeling and GAL4-directed expression of marker proteins, we analyzed the projections and compartmentalization of neurons expressing 12 different peptide genes, encoding approximately 75% of the neuropeptides chemically identified within the *Drosophila* CNS. Results are assembled into standardized plates which provide a guide to identify candidate afferent or target neurons with overlapping projections. In general, we found that putative dendritic compartments of peptidergic neurons are concentrated around the median Fas2 tracts and the terminal plexus. Putative peptide release sites in the ventral nerve cord were also more laterally situated. Our results suggest that i) peptidergic neurons in the *Drosophila* ventral nerve cord have separated in- and output compartments in specific areas, and ii) volume transmission is a prevailing way of peptidergic communication within the CNS. The data can further be useful to identify colocalized transmitters and receptors, and develop peptidergic neurons as new landmarks.

## Introduction

Neuropeptides are neuronal signaling molecules that are involved in the regulation of diverse processes such as development and growth, metabolism, reproduction, ion homeostasis, circadian rhythms and behavior. Neuropeptides can be produced by neurosecretory cells (secretory neurons) as well as interneurons, and are released as hormones into the circulation, or locally within the CNS. When released within the CNS, neuropeptides might act as “local hormones” via volume transmission (signal substance diffusion in a three-dimensional fashion within the extracellular space–also termed paracrine signaling), or as co-transmitters that act at or nearby synapses. In rare cases, neuropeptides have been identified in sensory cells and motor neurons of insects [1], [2].

Insect studies have revealed important insights into how neuropeptides and peptide hormones orchestrate behavior and integrate body functions [1]–[4]. In contrast, the role of direct synaptic input in the control of activity of peptidergic insect neurons is largely unexplored. This also applies to the fruit fly *Drosophila melanogaster*, although it has been shown that peptidergic *Drosophila* neurons express functional receptors for various neurotransmitters and biogenic amines [5]–[8]. The afferent pathways forming synaptic inputs on peptidergic neurons are still unidentified. The situation is similar regarding the output of peptidergic neurons. Although our knowledge on the behavioral or physiological effects of insect neuropeptides is steadily increasing (see [1]–[3]), the identity of targets of peptidergic neurons and the cellular mechanisms behind peptide signaling are commonly unknown, and we do not know whether centrally released peptides act as intrinsic or extrinsic neuromodulators (see [9]).

To answer questions concerning input and output relations of peptidergic systems, it is necessary to unravel the underlying neuronal circuitries. *Drosophila* is very well suited for this, owing to its relatively small number of neurons and its genetic amenability. Although the nervous system of *Drosophila* operates with a number of neurons that is around 100,000 times smaller than that of the primate brain, it seems to produce a similar diversity of neuropeptides. 31 neuropeptide genes have been identified so far [10]. These encode more than 60 putative peptides, 41 of which have been chemically identified within the CNS [10]–[13]. Concomitantly, the number of peptidergic neurons expressing a given peptide gene in the *Drosophila* CNS is very small (anything between two and several dozens of neurons), which allows us to individually identify peptidergic neurons. In combination with the available genetic tools, these features have made the fruit fly an established model organism to study neuropeptide signaling (see [14]).

Since the general organization of ventral ganglia is conserved throughout insects and crustaceans [15], unraveling the neuronal circuitry of peptidergic systems in the fruit fly can contribute to a general understanding of the architecture of peptidergic systems in the ventral nervous system and its neuroendocrinology in these most diverse animal groups. It may also provide insights into more general evolutionary design principles of neuropeptidergic systems, since recent findings suggest that not only the genetic control of neuroendocrine system development (see [16]), but also several peptide functions and signaling cascades bear significant homologies between *Drosophila* and vertebrates (e.g. [17]–[22]).

The aim of this study is to provide a detailed spatial description of the processes of peptidergic neurons in *Drosophila* and to identify possible in- and output compartments. By immunolabeling and GAL4/UAS-directed expression [23] of fluorescent marker proteins, we have analyzed the morphology and projections of neurons expressing 12 different peptide precursor genes. Our analysis focuses on the thoracic and abdominal neuromeres of the larva (for the sake of simplicity referred to as the larval ventral ganglion, which strictly speaking also comprises the suboesophageal neuromeres that are ignored here). This is mainly for two reasons, which may facilitate the identification of afferents and targets of peptidergic neurons: 1) evenly distributed landmarks of Fasciclin2 (Fas2) immunopositive tracts exist within the ventral ganglion neuropil that are constant between specimens and larval stages [24]. These landmarks allow us to describe projection patterns with high spatial accuracy in three dimensions. Since the Fas2 landmarks are used by several research groups to characterize neurite projections of e.g. sensory (e.g. [25], [26]) or motor (e.g. [24]) neurons, a comparison of the Fas2 “coordinates” of peptidergic processes with the projections of those neurons can provide a rationale to identify candidate afferents to or targets of peptidergic neurons, which in a second step can then be more rigorously analyzed. 2) The larval ventral ganglion is less complex than the brain, consists of a smaller number of neurons and shows in general a homomeric composition (see [27]). Despite its reduced complexity, the larval ventral ganglion nevertheless possesses peptidergic interneurons as well as neurosecretory cells producing peptide hormones, and receives sensory inputs of different modalities (see [27]).

As a basal step in deciphering peptidergic circuits, the data provides a morphological rationale to help identify candidate pre- and postsynaptic neurons by analysis of overlapping projections. Our results may additionally be useful for the identification of colocalized classical neurotransmitters, biogenic amines, peptides and receptors, and for developmental studies that rely on peptidergic neurons as landmarks.

## Results

**Figure 1:**
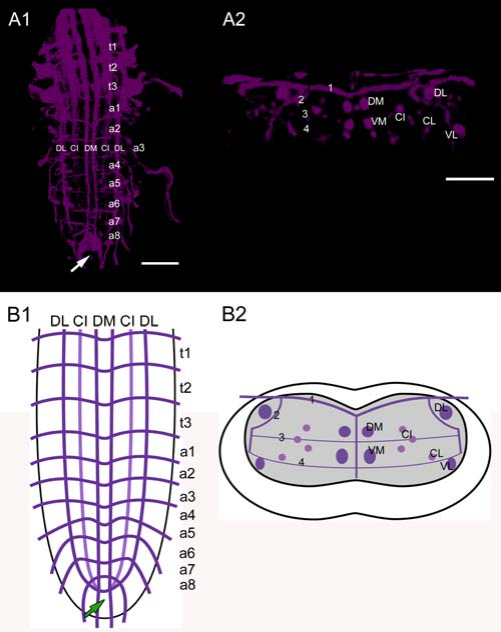
Fasciclin2 labeling. The Fas2 landmark system. A1) Dorsal view of a maximal projection showing longitudinal and transverse Fas2 immunoreactive fascicles. A2) Transversal view at the height of neuromere a3. B1) Idealized dorsal scheme B2) Idealized transverse scheme. Arrows in *A1* and *B1* show the neuromere a9 (“terminal plexus”). Scale bars: 50 µm in *A)*, 25 µm in *B)*.

In total, we have mapped peptidergic neurons expressing 12 different peptide precursor genes: *ast, capa, Ccap, cor, eh, Fmrf, hug, IFa, leucokinin, mip, pdf, and Dtk* (see table 1–2). This represents 32 processed peptides, or about 75% of the neuropeptides chemically proven to be produced by the CNS [10]–[13]. The peptide selection focused on neuropeptide hormones stored in thoracic or abdominal neurohemal organs (FMRFa-like peptides, CAPA peptides) or peripheral release sites on body wall muscles or the gut (CCAP, MIPs, leucokinin, PDF), and the availability of specific GAL4-lines. The results are presented in standardized plates (Fig. 1–13). Each plate shows a general overview of the observed immunolabelings or targeted mCD8.GFP expression in whole-mount preparations within the Fas2 landmark system. When considered useful, plates are accompanied by a supporting file (Fig. S1, S2, S3, S4, S5, S6, S7 and S8) which outlines further important details in a less rigorous way, including distribution patterns of ectopically expressed pre- and postsynaptic markers in case appropriate GAL4-drivers were available. The descriptions of the individual neuron labeling patterns below include a comprehensive comparison with previous morphological studies. This is preceded by a short summary of the known features of the respective peptides and their genes in *Drosophila*, and a comprehensive citation to published specificity tests of the antisera employed. The described projection patterns were highly constant throughout the preparations, whereas the position of somata was in general somewhat variable.

**Figure 2 pone-0000695-g002:**
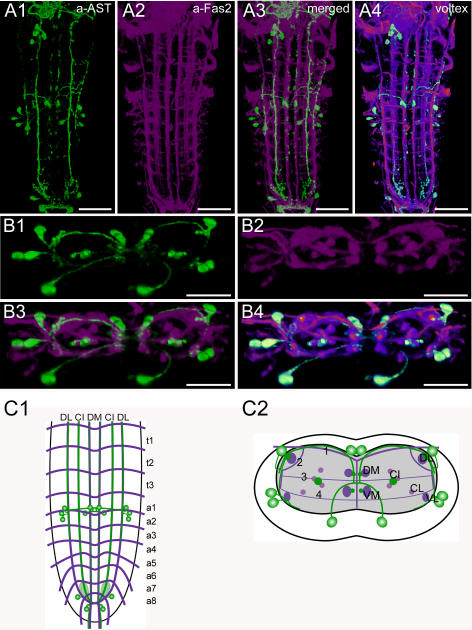
Morphology of AST-A neurons. Mapping of AST-A-IR neurons in whole-mount preparations of the thoracic and abdominal neuromeres of L3 larva in the Fas2 landmark system. A) Dorsal view. B) Transversal view at the height of neuromere a1/a2. C) Idealized dorsal scheme. D) Idealized transverse scheme. Scale bars: 50 µm in *A*), 25 µm in *B*). Immunostaining is shown in green, Fas2 in magenta.

**Figure 3 pone-0000695-g003:**
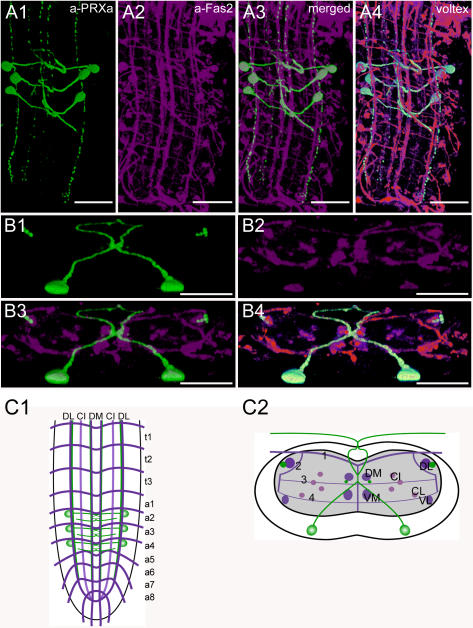
Morphology of CAPA- and HUGIN neurons. Mapping of PRXa-IR neurons in whole-mount preparations of the thoracic and abdominal neuromeres of L3 larva in the Fas2 landmark system. A) Dorsal view. B) Transversal view at the height of neuromere a3. C) Idealized dorsal scheme. D) Idealized transverse scheme. Scale bars: 50 µm in *A*), 25 µm in *B*). Immunostaining is shown in green, Fas2 in magenta.

**Figure 4 pone-0000695-g004:**
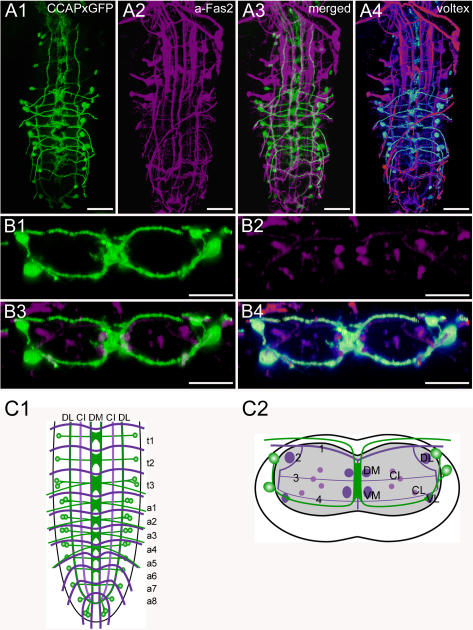
Morphology of CCAP neurons. Mapping of *Ccap*-GAL4xUAS-cd8.gfp expressing neurons in whole-mount preparations of the thoracic and abdominal neuromeres of L3 larva in the Fas2 landmark system. A) Dorsal view. B) Transversal view at the height of neuromere a3. C) Idealized dorsal scheme. D) Idealized transverse scheme. Scale bars: 50 µm in *A*), 25 µm in *B*). Marker protein expression is shown in green, Fas2 in magenta.

**Figure 5 pone-0000695-g005:**
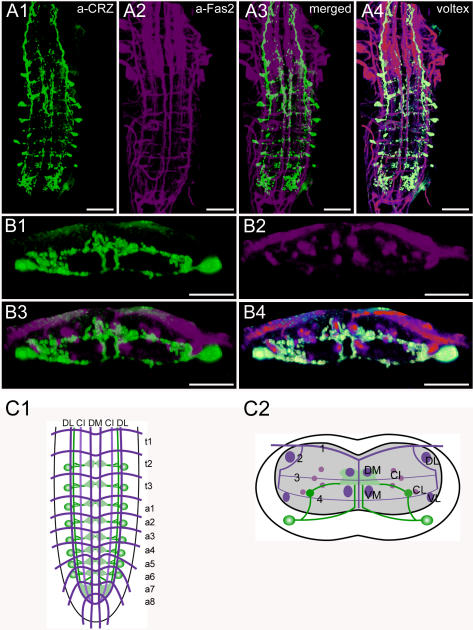
Morphology of corazonin neurons. Mapping of corazonin-IR neurons in whole-mount preparations of the thoracic and abdominal neuromeres of L3 larva in the Fas2 landmark system. A) Ventral view. B) Transversal view at the height of neuromere a6. C) Idealized dorsal scheme. D) Idealized transverse scheme. Scale bars: 50 µm in *A*), 25 µm in *B*). Immunostaining is shown in green, Fas2 in magenta.

**Figure 6 pone-0000695-g006:**
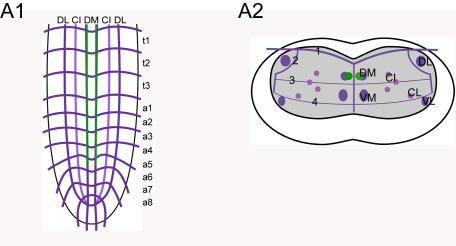
Morphology of eclosion hormone neurons. Mapping of *eh*-GAL4xUAS-cd8.gfp expressing neurons in whole-mount preparations of the thoracic and abdominal neuromeres of L3 larva in the Fas2 landmark system. A1) Idealized dorsal scheme. A2) Idealized transverse scheme.

**Figure 7 pone-0000695-g007:**
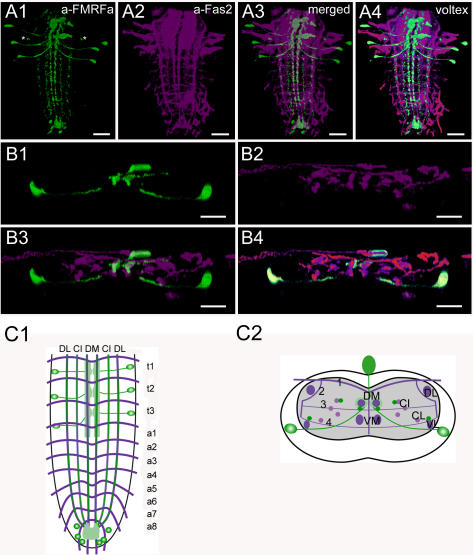
Morphology of FMRFamide neurons. Mapping of FMRFa-IR neurons in whole-mount preparations of the thoracic and abdominal neuromeres of L3 larva in the Fas2 landmark system. A) Dorsal view. B) Transversal view at the height of neuromere t2. C) Idealized dorsal scheme. D) Idealized transverse scheme. Scale bars: 50 µm in *A)*, 25 µm in *B*). Immunostaining is shown in green, Fas2 in magenta.

**Figure 8 pone-0000695-g008:**
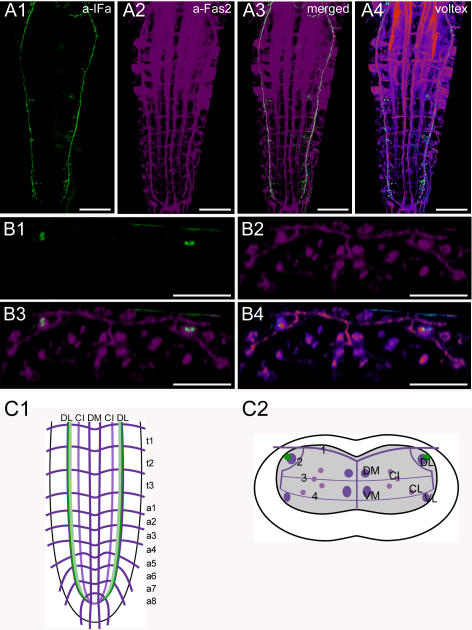
Morphology of IFamide neurons. Mapping of IFa-IR neurons in whole-mount preparations of the thoracic and abdominal neuromeres of L3 larva in the Fas2 landmark system. A) Dorsal view. B) Transversal view at the height of neuromere a3. C) Idealized dorsal scheme. D) Idealized transverse scheme. Scale bars: 50 µm in *A*), 25 µm in *B*). Immunostaining is shown in green, Fas2 in magenta.

**Figure 9 pone-0000695-g009:**
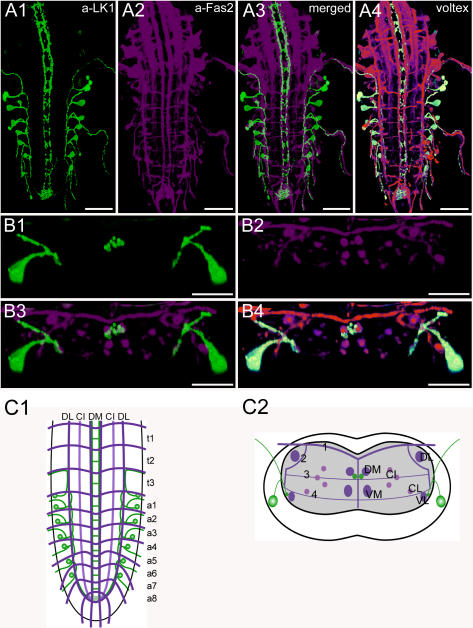
Morphology of leucokinin neurons. Mapping of leucokinin-IR neurons in whole-mount preparations of the thoracic and abdominal neuromeres of L3 larva in the FasII landmark system. A) Dorsal view. B) Transversal view at the height of neuromere a3. C) Idealized dorsal scheme. D) Idealized transverse scheme. Scale bars: 50 µm in *A*), 25 µm in *B*). Immunostaining is shown in green, Fas2 in magenta.

**Figure 10 pone-0000695-g010:**
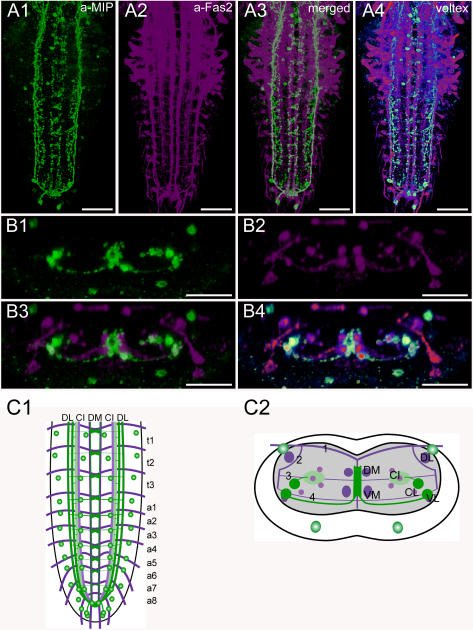
Morphology of MIP neurons. Mapping of Pea-MIP-IR neurons in whole-mount preparations of the thoracic and abdominal neuromeres of L3 larva in the Fas2 landmark system. A) Dorsal view. B) Transversal view at the height of neuromere a2. C) Idealized dorsal scheme. D) Idealized transverse scheme. Scale bars: 50 µm in *A*), 25 µm in *B*). Immunostaining is shown in green, Fas2 in magenta.

**Figure 11 pone-0000695-g011:**
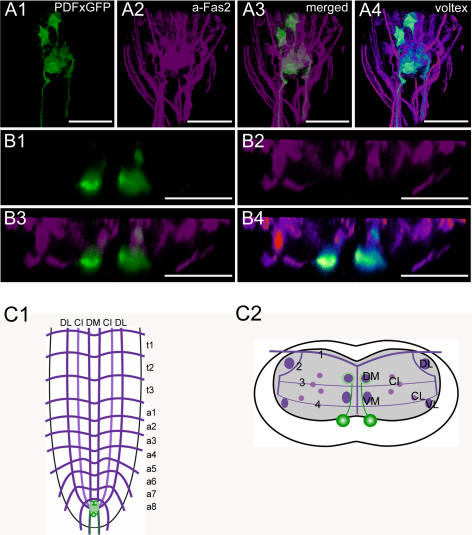
Morphology of PDF neurons. Mapping of *pdf*-GAL4xUAS-cd8.gfp expressing neurons in whole-mount preparations of the thoracic and abdominal neuromeres of L3 larva in the Fas2 landmark system. A) Dorsal view of the posterior part of the VNC. B) Transversal view at the height of neuromere a8. C) Idealized dorsal scheme. D) Idealized transverse scheme. Scale bars: 50 µm in *A*), 25 µm in *B*). Marker protein expression is shown in green, Fas2 in magenta.

**Figure 12 pone-0000695-g012:**
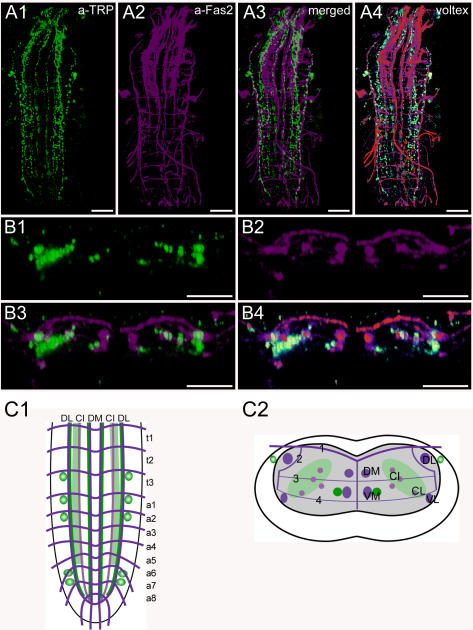
Morphology of tachykinin-related peptide-expressing neurons. Mapping of TRP-IR neurons in whole-mount preparations of the thoracic and abdominal neuromeres of L3 larva in the FasII landmark system. A) Dorsal view. B) Transversal view at the height of neuromere a1. C) Idealized dorsal scheme. D) Idealized transverse scheme. Scale bars: 50 µm in *A*), 25 µm in *B*. Immunostaining is shown in green, Fas2 in magenta.

**Figure 13:**
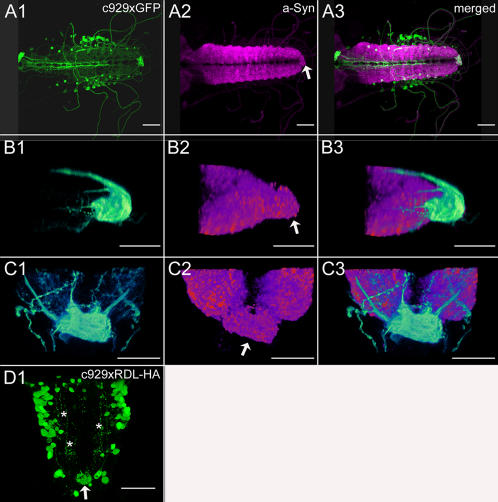
Distribution of c929-GAL4 driven expression of synaptic markers. *A*) Dorsal view of the VNC of a c929-GAL4xUAS-GFP larva, voltex projection. *B*) Lateral view of a8–9 of a c929GAL4xUAS-GFP larva, voltex projection. *C*) Dorsal view of a8–9 of a c929GAL4xUAS-GFP larva, voltex projection. Note the reduced morphology of the neuropil in a9 ( = the terminal plexus), immunolabeled against the synaptic marker protein synapsin (arrow) in *A–C*). *D*) Dorsal view of a c929GAL4xUAS-RDL.HA larva, maximum projection. RDL.HA immunostaining labels the cell bodies, descending neurites (asterisks) and the terminal plexus (arrow). Scale bars: 100 µm in *A*), 20 µm in *B–C*), 50 µm in *D*). HA-immunostaining or GFP expression is shown in green, synapsin-IR in magenta.

**Table 1:** Employed fly lines

Lines	Donor [Reference]
*pdf-*Gal4	Jeff Hall [119]
*Ccap-*Gal4	John Ewer [42]
*Eh-*Gal4	John Ewer [51]
Va-Gal4	Stefan Thor [40]
*hug-*Gal4	Michael Pankratz [35]
c929-Gal4	Paul Taghert [78]
*fmrf-*Gal4	Paul Taghert
UAS-cd8.gfp	Bloomington Stock Center, Tzumin Lee and Liqun Luo [120]
UAS-*nls*.*RedStinge*r	Bloomington Stock Center, Jim Posakony
UAS-*n-syb.egfp*	Bloomington Stock Center, Kendal Broadie [88]
UAS-*rdl.HA*	Andreas Prokop [89]

**Table 2:** Employed antisera

Antisera/mAb	(Dilution) Source	Donor [Reference]
Anti-CRZ	(1∶2000) rabbit	Jan Veenstra [49]
Anti-CCAP	(1∶1000) rabbit	Heinrich Dircksen [44]
Anti-FMRFa	(1∶4000) rabbit	Eve Marder [63]
Anti-IFa	(1∶500) rabbit	Peter Verleyen & Liliane Schoofs [65]
Anti-LK-1	(1∶2000) rabbit	[68]
Anti-MIP	(1∶4000) rabbit	Manfred Eckert [71]
Anti-PDF	(1∶4000) rabbit	[76]
Anti-PRXa	(1∶4000) rabbit	Manfred Eckert [39]
Anti-TRP	(1∶4000) rabbit	[121]
Anti-allatostatin-A	(1∶5000) rabbit	Hans Agricola [32]
anti-Fas2 mAb 1D4	(1∶75) mouse	Developmental Study Hybridoma Bank, Corey Goodman
anti-HA mAb 3F10	(1∶75) rat	Roche Diagnostics, Mannheim, Germany

The neutral nomenclature of the Fas2-positive tracts (Fig. 1, Video S1, S2, and S3) follows Landgraf et al. [24], who also provide a general morphological description. Longitudinal tracts are designated by letters relative to their position in the dorsoventral (D, dorsal; C, central; V, ventral) and mediolateral (M, medial; I, intermediate; L, lateral) position. The five transverse Fas2-positive projections are numbered 1–5 according to their dorsoventral position. The DI and VI tracts were omitted in our schematic diagrams, since they were not always distinguishable and their inclusion would not have added to a more precise localization of peptidergic neurites. The metameric Fas2-staining relates to the segmental neuromeres as follows: each segmental nerve of the abdominal neuromeres a1–a7 contains two tracts, the posterior segmental nerve (SN) which originates from the neuropil of the homotopic neuromere, and the anterior intersegmental nerve (ISN). The ISN consists of an anterior root (aISN) originating from the posterior commissure (pC) of the immediately anterior neuromere, and a posterior root (pISN) originating from the anterior commissure (aC) of the homotopic neuromere [27]. The most prominent Fas2-IR transversal projection visible in the dorsal views is TP-1 which equals the pISN in the late larval stage [24]. Thus, the segmental borders are somewhat anterior to TP-1. Whereas the organization of the thoracic neuromeres t1–t3 is very similar to that described for a1–a7, there are differences in the segmental nerves of the last two abdominal neuromeres belonging to the tail region. The nerve of a8 only comprises a single fascicle deriving from three roots; the segmental nerve of a9 only has two roots [27].

In the figures, the CNS always appears flattened. This is due to slight compression of the preparations caused by the paraformaldehyde fixation, and due to the shifting light refractive index that is unavoidable during confocal z-scans (see [28]). These effects were not corrected, since they apply alike to Fas2 tracts and peptidergic (and other) neurons and vary depending on the thickness of the preparations. It is thus an advantage of relative landmark systems that these artefacts do not influence the overall results, since the morphological description is only relative to the Fas2 tracts.

### Morphology of AST-A neurons (Fig. 2)

In *Drosophila*, three genes [*ast* (CG13633), *mip* (CG6456), *ast2* (CG14919)] encode precursors of peptides that have been designated allatostatins (AST): AST-A, B and C. This is unfortunate since they do not all display allatostatic activity in a given insect species. AST-B peptides are also commonly referred to as myoinhibitory peptides (MIPs) and will be described in a later section. Here, we analyze the distribution of AST-A peptides (Drostatin-A1–4) derived from the *ast* precursor [29]. The four peptides are processed as predicted from the genome in the larval and adult CNS [11], [13], but their functions are still unknown. In other insects, Ast-A peptides show either allatostatic or myoinhibitory activity (see [30]).

The distribution pattern of AST-IR neurons (AST neurons) was described by Yoon and Stay [31] with a mouse mAb to *Diploptera* AST-1. We have reproduced this labeling pattern with a polyclonal antiserum against *Diploptera* AST-1 [32] and therefore adopted the nomenclature of Yoon and Stay. Since the polyclonal antiserum recognizes different *Dip*-allatostatins sharing the allatostatin A consensus sequence YXFGLa [32], we assume that the immunoreactivity in *Drosophila* represents at least Drostatin-A1-3 possessing a C-terminal YXFGLa.

The ventral ganglion contains 8 pairs of AST neurons, all located in the abdominal neuromeres. In the first abdominal neuromere (a1), a bilateral pair of AST neurons is located dorsomedially (DMA cells) in the cortex right above the DM tracts and the bifurcation point of transversal projection TP1 (1). Another pair of AST neurons resides in the ventromedial cortex (VMA cells); the position of their somata varies between the VM and the CI tracts. Furthermore, three pairs of neurons are arranged laterally in a1–2. One pair of somata resides dorsally nearly at the height of the DL tract (DLA cells); the other two adjacent pairs are located more ventrally at the height of the VL tract (VLA cells). The last abdominal neuromeres (a8/9) include three pairs of AST neurons (DLAa cells). One pair is located laterally in a8, whereas the other two are arranged more medially in a9, nearly at the tip of the ventral ganglion.

Longitudinal axonal projections of AST neurons are adjacent to the DM, VM, CI and DL tracts. The AST projections close to the DM, VM and DL tracts are poorly stained in a4–a8, whereas those close to the CI tracts are most prominently stained. The longitudinal axon projections adjacent to the CI tracts converge in the neuropil of a8, where they give rise to arborizations with pronounced varicosities. Due to the strong staining of the axon projections following the CI tracts, and their varicose endings in a8, it seems likely that these structures represent a major release site of AST peptides, whereas the weaker stained AST projections close to the DM and VM tracts might represent dendritic areas containing fewer peptidergic vesicles.

In the neuropil, the axons of the DMA, VMA, DLA, VLA and DLAa cells converge closely with longitudinal AST projections, which made it impossible to follow their individual paths in detail. The DMA neurons project ventrally and seem to join longitudinal AST projections between the DM and the VM tracts. Each VMA cell sends a single axon dorsomedially, first passing between the DM and VM tract, and then joining with the contralateral neurite in the midline somewhat below the TP3 tract. There, the axons appear to cross to the contralateral side and continue together dorsally before they diverge in the neuropil beneath the DMA cells and project laterally above the TP1 tract. In the direct neighborhood of the DL tract, the VMA axons bilaterally join the longitudinal AST projections. These longitudinal AST projections are also connected to the DLA and VLA cells. Whereas each of the short axons of the DLA cells forms a small ventral loop before contacting the longitudinal AST projection close to the DL tract, the axons of the VLA cells project dorsally and join the same longitudinal AST projections behind the axons of the VMA and DLA cells. The DLAa cells of a8/9 extend their axons medio-anteriorly where they join with the longitudinal AST projections along CI and proceed posteriorly to exit in the last segmental nerve. These efferent axons innervate the surface of the hindgut [31].

### Morphology of *capa*- and *hugin* neurons (Fig. 3, S1)

The *hugin* (*hug*) precursor gene (CG6371) encodes a pyrokinin (HUG-PK [33]). The *capability* (*capa*) gene (CG15520) encodes a prepropeptide containing two periviscerokinins (CAPA-PVKs) and one pyrokinin (CAPA-PK) [34]. All of these peptides share the C-terminal sequence PRXa and are processed as predicted from the genome; CAPA-PK also occurs in a shortened form CAPA-PK^2–15^ in the suboesophageal neuromeres and in the ring gland [11]–[13]. A further potential PRXa (HUG-γ) is encoded by the HUGIN prepropeptide, but has not been shown to be processed. The *hug* gene has been implicated in regulation of feeding behavior in relation to chemosensory and nutritional signals [35] and CAPA-PVKs display diuretic activity on Malpighian tubules [34].

The pattern of PRXa-expressing neurons (PRXa neurons) in *Drosophila* was described using polyclonal antisera raised against Hez-pheromone biosynthesis activating neuropeptide, *Aplysia* myomodulin, and Pea-CAPA-PVK-2 [36]–[38]. The distribution of HUG-PK was studied by *in situ*-hybridization and by *hug*-GAL4-directed GFP-expression [35]. The expression of CAPA was described by *in situ* hybridization and a precursor-specific antiserum [34]. In this study, we have used a polyclonal antiserum against Pea-PVK-2, which specifically recognizes the C-terminus PRXa ([39]) shared by the CAPA and HUGIN peptides, as well as marker molecule expression driven by Va-GAL4 (see below, [40]) and *hug*-GAL4 [35]. The ventral ganglion contains 3 pairs of strong PRXa-IR neurons (Va neurons): a bilateral pair of these Va neurons is located ventrally in each of the first three abdominal neuromeres. The Va neurons express *capa* and process all three CAPA peptides [12], [34]. The position of the Va somata varied between preparations from a more median to a more lateral ventral position close to the ventral lateral (VL) tract. Typically, the somata on each side are arranged in a row. Each cell body sends a single axon dorsomedially through the neuropil, first passing below the central intermediate (CI 1–3) longitudinal Fas2-IR tracts, and then below the dorsal median (DM) tracts. Each axon reaches the midline at about transverse projection 3, where it joins with the axon of the contralateral Va neuron. The axons subsequently project together a short distance dorsally before they diverge and form a loop inside the neuropil and dorsal cortex. The loop closes just before the neurites leave the ventral ganglion and pass through the median nerve to finally enter the transverse nerves, where they end blindly. The median nerve and the innervated proximal part of the transverse nerves in a1–3 are neurohemal release sites (perisympathetic organs) for the CAPA peptides [38]. Va-GAL4-driven SYB.EGFP only labels the Va somata where the protein is produced, as well as the median nerve and the proximal part of the transverse nerves, but not the Va axons (Fig. S1). The accumulation of the vesicle marker SYB.EGFP fits well with the neurohemal function of the perisympathetic organs. Interestingly, we could not find morphological correlates for arborizations within the CNS by either PRXa immunostainings, or by GFP-expression and subsequent anti-GFP staining.

Descending PRXa-IR neurites run along each DM and DL tract and terminate in a7 around the border to a8. These neurites contain HUG-PK as shown by PRXa immunostaining of *hug*-GAL4xUAS-GFP flies.

### Morphology of CCAP neurons (Fig. 4, S2)

The CCAP prepropeptide is encoded by the *Ccap* gene (CG4910). In *Drosophila*, CCAP has not been detected biochemically so far. In *Drosophila*, CCAP has an important role in the regulation of heart beat and ecdysis-related behaviors [41], [42].

The distribution pattern of CCAP neurons has been described by immunostainings with a polyclonal antiserum produced by HJ Agricola [42], [43], and by *in situ* hybridization as well as a specific *Ccap*-GAL4-line [42]. In our study, we relied on a specific polyclonal antiserum produced and characterized by H Dircksen [44] as well as the *Ccap*-GAL4 line.

The observed pattern of *Ccap*-GAL4-driven GFP-expressing neurons (CCAP neurons) matched generally that of the known patterns of CCAP-immunoreactivity [43] and *Ccap*-expression [42], though there are a few differences in the number of neurons and in the extent of GFP-expressing neurites [8]. In t1 and t2, a bilateral pair of CCAP neurons is located ventrolaterally, i.e. nearly at the height of the VL tract. In contrast, t3 and a1–4 each contain two pairs of CCAP neurons. These neurons are also located laterally. The position of their somata in a1–4 varied in the dorso-ventral axis from a more median to a more dorsal position, i.e. between the height of the VL and the DL tract, whereas the pairs in t3 are ventrally located (Fig. S2). In a5–7, only one pair of lateral neurons expresses *Ccap*-GAL4-driven GFP; their somata reside between the height of the VL and the DL tract. We could however visualize single additional CCAP neurons by immunostaining in a5–7. Thus it seems likely that also a5–7 contain two pairs of CCAP neurons. The last abdominal neuromeres a8–9 contain three pairs of CCAP neurons, one is located laterally, whereas the other two are arranged more medially, nearly at the tip of the ventral ganglion. In many preparations, GFP-expression in these CCAP neurons of a8–9 is weak. Single descending CCAP neurites project very close to the VL tracts on both sides and coincide in the terminal plexus of a9. Further descending neurons, most probably originating in the brain, run along the DM fascicles (Fig. S2).

Each CCAP neuron in t1–3 and a1–7 sends a neurite beneath the transversal projection 4 ventromedially, where the neurites from both sides join in proximity to the VM tracts. These joining CCAP neurites form extensive arborizations along the midline between the DM and VM tracts, which are most prominent in t3. In t3 and a1–4, the medially joined neurites of at least one neuron pair project further dorsally until they leave the central neuropil. These neurites diverge in the dorsal cortex, project via the segmental nerves to the periphery, and form type III terminals on body wall muscles M12 and M13 [8].

Based on release studies with a peptide-GFP construct, the descending neurites along the VL tract have been suggested to be central CCAP release sites [45]. This suggestion is supported by the accumulation of CCAP-IR material and *ccap*-GAL4-driven SYB.EGFP in these descending neurites. Furthermore, both CCAP-IR and SYB.EGFP, but not GFP, strongly label median descending neurites with pronounced varicosities along the DM fascicles. This suggests that CCAP is also released along the DM tract.

In contrast, the putative postsynaptic marker RDL.HA (see discussion) is exclusively present between the DM and VM tracts at the region of the extensive arborizations of the thoracic and abdominal CCAP neurons (Fig. S2). These arborizations are strongly labeled by GFP, only faintly labeled by SYB.EGFP, and unlabeled in CCAP immunostainings (Fig. S2). We thus assume that the median arborizations around the DM and VM tracts represent a dendritic compartment, whereas CCAP release mostly occurs from descending neurites along the VL and DM tracts as well as from peripheral release sites on muscle M12 and M13 [8].

### Morphology of corazonin neurons (Fig. 5, S3)

The *Drosophila* corazonin prepropeptide is encoded by the *crz* gene [46], [47]. Corazonin occurs in full length, but also in a shortened form, corazonin^3–11^ [11]–[13]. The function of corazonin in *Drosophila* is so far unknown, although it might be associated with the circadian clock and the control of ecdysis [47], [48].

The pattern of corazonin-IR neurons (corazonin neurons) in *Drosophila* was described by Choi et al. [47] with a specific polyclonal antiserum as well as *in situ* hybridization, and by Landgraf et al. [24] with a polyclonal antiserum produced by J Veenstra [49]. In this study, the Veenstra antiserum was used and yielded results identical to those previously described.

In total, 16 corazonin neurons are located in the ventral ganglion. In t2–3, and a1–6, one bilaterally symmetric pair of strongly stained neurons was observed. Each soma is located ventro-laterally approximately below the VL tract. It sends a neurite towards the midline which bifurcates shortly after leaving the soma. The lower branch projects ventrally towards the midline, then bends dorsally and enters the neuropil below and slightly medial to the VM tract. At the dorsal height of the VM tracts, it enters a medial varicose region located between the DM and VM tracts. The upper branch first projects dorsally until it reaches a longitudinal corazonin-IR tract that runs along the inner side of the CL tract. It then bends medially between the CI tracts until it reaches the varicose region between the DM and VM tracts. This varicose region seems to be formed by endings of both upper and lower branches. The longitudinal corazonin-IR tract along the CL tract extends posteriorly until a7, and anteriorly into the brain. At both endings, the tract arborizes and forms a varicose field. In the ventral ganglion, this field fills the neuropil between DL/VL and DM/VM tracts of a7–8, is strongly immunoreactive and extends ventrally from an intermediate position to the VM tracts (see also Fig. S3). The longitudinal corazonin-IR fascicle appears to be formed by neurites of the corazonin neurons in the ventral ganglion. It was, however, impossible to distinguish whether only some or all corazonin neurons contribute to it. Whereas the complete fascicle could be visualized with the corazonin antiserum in the thoracic neuromeres, staining broke up into varicosities in the abdominal neuromeres. Since varicosities are putative peptide release sites, it appears that corazonin is released from the longitudinal corazonin-IR fascicle along the CL tract in the abdominal neuromeres, the median neuropil between the DM and VM tracts, and especially within the neuropil of a7/a8.

### Morphology of eclosion hormone neurons (Fig. 6)

The eclosion hormone (EH) is encoded by the *Drosophila* gene *Eh* (CG5400 [50]) and plays an important role in the regulation of ecdysis behavior [51]. It has so far not been chemically characterized in *Drosophila*.

The morphology has been described by immunostaining [50], and with an *Eh*-GAL4 line [51] that was also used in this study.

EH is exclusively expressed in a single pair of neurons with somata in the ventromedial brain. Their axons (one per hemisegment) descend along, and to some extent overlap, the DM tract and terminate at segment A5/A6. In our hands, the *Eh-*GAL4 line was only a weak driver for marker molecules, and we could only label somata by ectopic expression of RDL.HA or SYB.EGFP.

### Morphology of FMRFamide neurons (Fig. 7, S4)

The *Drosophila Fmrf* precursor gene (CG2346) encodes eight different FMRFa-like neuropeptides [52]–[54], all of which have been chemically identified in the CNS [11]–[13]. In *Drosophila*, FMRFa-like peptides may play a role as hormones during ecdysis and myomodulators on body wall muscles and the heart [55]–[57].

The larval pattern of FMRFa-IR neurons (FMRFa neurons) was described with different polyclonal antisera raised against FMRFa [53], [54], [58]–[60], and by *in situ* hybridization [53], [61]. In this study, we employed a *fmrf*-GAL4 driver [62] to express GFP, and a polyclonal anti-RFamide serum [63] that is expected to label FMRFa-like peptide 2–7 and probably also other peptides containing RFamide in its C-terminus in *Drosophila*.

The overall pattern of the 14 FMRFa-IR neurons is basically similar to that described in earlier studies [53], [54], [58], [59], although several median FMRFa-IR neurons reported in these studies could not be found in our immunostainings. The nomenclature of neurons follows [54]. In each of the thoracic neuromeres, one pair of strongly staining Tv neurons could be visualized, which were also included in the *fmrf-GAL4* driver line. Their somata are quite large and elongated, located very close to the lateral border of the neuromeres ventrolaterally to the VL tracts. Each Tv neuron sends a single axon toward the midline. It first projects below the VL tract, bends upward below the CL and CI tracts, and approaches the DM tract from below. Strongly GFP-labeled arborizations were visible around the DM tract. These arborizations are probably dendritic input areas, since they were not marked by *fmrf*-GAL4-driven expression of SYB.EGFP (Fig. S4). In the anterior-posterior axis, the dendritic areas of the three pairs of Tv neurons form a continuous band from t1 to about half the way between a1 and a2. The axon of each Tv neuron then fasciculates with that of the contralateral Tv neuron and projects into the respective neurohemal thoracic perisympathetic organ (tPSO) where strong arborizations and varicose structures were visible. The tPSOs exhibited a strong accumulation of *fmrf-GAL4*-driven SYB.EGFP (Fig. S4), indicating that FMRFa-like peptides are stored and released from the tPSOs. One smaller neuron in the second thoracic neuromere near each Tv soma was also FMRFa-IR (asterisk in *A1*); its neurites could however not be visualized. In the terminal abdominal neuromere a9, 6 At1 cells could be stained. Again, their neurites could not be visualized.

On each site of the ventral ganglion, three descending varicose FMRFa-IR neurites that run medial along the CI tract, ventral to the DM tract, and dorsal to the VL tract. These FMRFa-IR neurites terminated in the neuropil of the terminal abdominal neuromere which showed strong FMRFa-immunoreactivity.

### Morphology of IFamide neurons (Fig. 8)

The *IFa* gene (CG33527) of *Drosophila* encodes one IFamide (IFa) which is processed as predicted from the genome [11], [13]. IFa is a potent modulator of sexual behavior in the fruit fly [64].

The pattern of IFa-IR neurons (IFa neurons) has been described with a polyclonal antiserum against the LFamide of the flesh fly *Neobellieria bullata* [65], and by immunostaining and *in situ* hybridization [64]. In this study, the *Neobellieria* LFamide antiserum was used and yielded results identical to those described.

In the ventral ganglion, IFa-IR somata (IFa somata) are absent. However, two pairs of IFa somata are strongly stained in the anterior medial region of the larval brain. These neurons send descending axons (one in each hemisphere) along the DL fascicles through the complete ventral ganglion. These IFa axons end abruptly in a8/9, just before the longitudinal Fas2 fascicles terminate at the border of the terminal plexus of a9. Arborizations within the terminal plexus were never observed. Varicosities are pronounced along short side-branches oriented medially towards the neuropil in each of the thoracic and abdominal neuromeres. These varicosities suggest that IFa is released along the whole DL tract within the thoracic and abdominal neuromeres.

### Morphology of leucokinin neurons (Fig. 9, S5)

The *Drosophila Leucokinin* precursor gene (CG13480) encodes one leucokinin [66], which is processed as predicted from the genome [11], [13], [66]. Leucokinin acts as a diuretic hormone on the Malpighian tubules [66].

The pattern of leucokinin-IR neurons (LK neurons) was described in *Drosophila* [24], [67] with a polyclonal antiserum raised against *Leucophaea* leucokinin I [68]. In this study, we reproduced this pattern using the same antiserum.

In each of the first seven abdominal neuromeres, one bilateral pair of strongly stained neurons was observed. In general, the most anterior neurons appeared to be gradually larger and were placed closer to the external cortex border than the posterior neurons. Each soma is located ventro-laterally in each hemi-neuromere approximately at the height of the VL tract. It sends a neurite dorsomedially that divides into two branches just before or when entering the neuropil at the height of the lower CI fascicles. The dorsal branch projects further dorso-anteriorly, leaves the ventral ganglion through the segmental nerve of the next anterior segment (segmental nerves of t3 and a1–6), and forms peripheral release sites on body wall muscle M8 [67].

The second branch turns ventrally until it reaches the VL tract where it seems to become a part of a descending leucokinin fascicle along the VL tract from a1 to a8. The leucokinin-fascicle enters the neuropil of a9 (the “terminal plexus” [24]), where it strongly arborizes. The terminal plexus is also innervated by two descending varicose leucokinin fascicles that are associated with the DM tracts. These descending neurites seem to originate from somata in the first suboesophageal ganglion [24], [67].

Based on the distribution of varicosities, two putative release areas of leucokinin can be postulated: peripheral release sites of the abdominal leucokinin neurons mostly on muscle M8 [67], and central release sites along the DM tract throughout the whole ventral ganglion originating from leucokinin neurons in the first suboesophageal neuromere.

### Morphology of MIP neurons (Fig. 10)

The *Mip* prepropeptide gene (CG6456) of *Drosophila* encodes 5 putative myoinhibiting peptides (MIPs [69], also designated AST-B-1-5 [70]). Three of these have been chemically characterized within the CNS [11], [13]. The functions of MIPs in *Drosophila* are unknown, although they are likely to play a role during ecdysis [57].

The distribution of MIP-expressing neurons (MIP neurons) has been studied by *in situ* hybridization and by immunostaining [57], [70]. Kim et al. [57] assigned two pairs of MIP neurons co-expressing CCAP to the abdominal neuromeres a1-4 and a8/9, whereas MIP neurons seemed to be absent in a5–7. Neurons that lacked CCAP-expression but showed MIP-IR (e.g. in the thoracic neuromeres and in a8/9) were not described. We have employed a polyclonal antiserum directed against Pea-MIP (GWQDLQGGWa) [71], yielding slightly different results. We assume that the polyclonal antiserum recognizes MIP-1 (AWGSLQSSWa) and MIP-5 (DQWQKLHGGWa), if not all *Drosophila* MIPs which share the C-terminal Wamide.

Two Pea-MIP IR neurons (Pea-MIP neurons) are arranged at each side in two different bilateral symmetric rows in neuromeres t1–a7. The first row of Pea-MIP neurons is located dorso-laterally nearly at the height of the DL tract (at least some of these neurons co-express CCAP), the second row resides ventromedially at the width of the CI tracts. In a8/9, four pairs of Pea-MIP neurons are grouped at the tip of the ventral ganglion. Longitudinal projections of Pea-MIP neurons are adjacent to the DM, VM and VL tracts, and above the Cl tract. All longitudinal Pea-MIP projections coincide in the terminal plexus of a9, forming a small terminal area with many varicosities. The Pea-MIP longitudinal projection adjacent to the Cl tract innervates an area with many varicosities around the CI tracts. Transversal projections of Pea-MIP neurons reside ventro-medially at the height of the transverse tract 4. The transversal Pea-MIP neurites from both sides join with the central longitudinal Pea-MIP projections in proximity to the VM tracts and establish extensive arborizations in the midline of the central neuropil between the DM and VM tracts.

### Morphology of PDF neurons (Fig. 11, S6)

In *Drosophila*, pigment-dispersing factor (PDF) is encoded on the *Pdf* gene (CG6496, [72]) and processed as predicted from the genome. In the brain, PDF has important roles within the circadian system (e.g. [73]).

The pattern of larval PDF-IR neurons (PDF neurons) was described with a polyclonal antiserum raised against crustacean β-PDH [44], [74], [75]). In this study, we could reproduce this pattern using a characterized polyclonal antiserum produced against *Drosophila* PDF [76], [77], with hitherto undescribed dendritic arborizations that were visualized by *pdf*-GAL4-directed GFP-expression as described below.

PDF neurons occur in two groups: one consisting of 3–4 somata in a8, and another consisting of 4 somata in a9. These numbers are based on the *pdf-GAL4*-driven expression of a nucleus-targeted dsRed-variant, since the cell number was difficult to assess with immunolabelings or membrane-targeted GFP. The somata in a8 are located ventrally directly below the neuropil region, slightly lateral below the VM tracts. Neuromere 9 does not show the typical Fas2-positive pattern. Therefore, a scheme is not given, and positions are described with reference to the Fas2-positive tracts in a8. The somata in a9 are more dorsally located then those in a8, just behind the prominent posterior neuropil at about the height of the DM tracts. The medio-lateral position of the somata in a9 is similar to that in a8, i.e. slightly lateral of the DM/VM tracts. Unlike other peptidergic cell bodies, the PDF somata show a rhombic or polygonal shape. The PDF neurons send immunostained axons through the 8th abdominal segmental nerve into the periphery. Closer analysis of the distribution of *pdf-GAL4* driven mCD8-GPF reveals pronounced arborizations in the terminal plexus, the neuropil of a9. Anterior to the terminal plexus, *pdf*-GAL4-driven GPF was also visible along the DM tracts in A8. Since these arborizations in the terminal plexus and along the DM tracts are not labeled by different antisera against PDF ([74], this study) and *pdf*-GAL4-driven SYB.EGFP (Fig. S6), we regard these arborizations to be the PDF neuron dendrites. Although PDF is an amidated peptide, the abdominal PDF neurons are not included in L3 larvae of the c929-*GAL4* line specific for neurons expressing the amidating enzyme PHM ([78], not shown).

Besides neuromeres 8 and 9, PDF labeling is absent from the rest of the VNC, descending projections from the suboesophageal ganglion or the brain were not observed.

### Morphology of TRP neurons (Fig. 12, S7)

The *Drosophila* gene *Dtk* (CG14734) encodes a prepropeptide containing 5 tachykinin-related peptides, DTK1-5 [79], all of which are expressed in the brain [80]. In the fruit fly, DTKs have a modulatory role in olfactory perception and locomotor activity [81].

The distribution of tachykinin-related peptide-IR neurons (TRP neurons) has been described by *in situ* hybridization and immunostaining with a polyclonal antiserum against the cockroach LemTRP-1 (APSGFLGVRamide) [79], [80]. We used the same antiserum and found a pattern similar to that described in [80], although the reported weakly TRP-IR neurons expressing *Dtk* could not be visualized in our immunostainings. We assume that the immunoreactivity recognizes all DTKs which share the C-terminal sequence FXGXRamide with LemTRP-1.

In the ventral ganglion, five pairs of neurons were immunostained: one pair in t3, and one pair each in a1, a2, a6 and a7. As in the earlier studies, we could not visualize the neurites of these neurons.

Several TRP-IR neurites with prominent varicosities descend from the brain and suboesophageal ganglion. One pair of TRP-IR neurites descend along the midline parallel to the lateral side of the DM tracts until T3. In some cases, these TRP-IR neurites extended as far as A2, or even further. In most preparations, a second neurite descends from T3 along the outer side of the VM tracts up to a8 on each side. In a few cases, these longitudinal neurites descended from T2. Moreover, a broad varicose region is formed by descending neurites from the brain that are situated mostly between the VL and the CI tracts up to a8. Further lateral TRP-IR neurites descend along the DL tract and terminate in a7 or a8.

On each side in T3, a TRP-IR fascicle runs transversely ventral from the DL tract, continues through the CI-tracts and meets the fascicle from the other side at the midline between the DM and VM tracts (Fig. S7). These fascicles seem to originate from the TRP-IR somata in T3.

Based on the assumption that strongly varicose regions indicate peptide release sites, all the described descending neurites might represent peptide release sites with a particular concentration in the area between the CI and VL tracts.

### Distribution of ectopical c929-GAL4-driven SYB.EGFP and RDL.HA (Fig. 13, S8)

The c929-GAL4 line (*dimmed*) is specific for peptidergic neurons and specifies around 200 neuroendocrine cells/neurons, i.e. a large proportion of the peptidergic neurons [78]. c929-GAL4-driven expression of GFP, the presynaptic marker SYB.EGFP, and the postsynaptic marker RDL.HA labeled the same peptidergic neuron somata, and matched the previously described pattern [78]. In contrast, differences in the distribution of the marker proteins were found within neuropil areas (Fig. S8). GFP fluorescence was most intense in the terminal plexus of a9, median fascicles around the DM tracts in the thoracic and the first 3–4 abdominal neuromeres, and lateral fascicles along the DL/VL tract. SYB.EGFP fluorescence occurred in a punctate fashion, in accordance with its suspected localization in the membrane of peptidergic vesicles accumulating at release sites. Spots of high SYB.EGFP fluorescence are thus likely to represent vesicle storage or release sites. SYB.EGFP fluorescence was most intense in the thoracic PSOs, the lateral fascicles and throughout the neuropil of a7/8, suggesting these structures as prominent peptide release sites. The terminal plexus of a9, and the median fascicles contained lesser amounts of SYB.EGFP fluorescence, but also here SYB.EGFP presence suggests that peptides are released at these structures. Noteworthy, also an intermediate fascicle expressing SYB.EGFP was visible, a structure that could also be labeled with lacZ [78], but was not prominently labeled by GFP. The most restricted distribution pattern in the neuropil was found for c929-GAL4-driven RDL.HA, which was concentrated within the terminal plexus of a9 (Fig. 13). Strong contrast enhancement of the voltex projections revealed further RDL.HA localization at lateral, intermediate and median projections (Fig. 13).These results suggest that the terminal plexus contains a higher density of peptidergic postsynaptic sites of peptidergic neurons than any other neuropil area. Postsynaptic compartments of peptidergic neurons appear, however, also to be located at lateral, intermediate and median peptidergic fascicles. Since the anti-RDL.HA staining intensity in general was very weak, areas with a low density of peptidergic postsynapses might have escaped our analysis.

## Discussion

Based on our comparative analysis of the projection patterns of processes from different peptidergic neuron types within the Fas2 landmark scaffold, some broader generalizations about the morphological organization of peptidergic systems within the ventral ganglion can be made. These are discussed in the following sections.

### Serial homology

As in other eumetazoans, the insect body is segmentally organized. The general organization of thoracic and abdominal ganglia is similar and well preserved throughout the insects and crustaceans [15]. Serial homology (i.e. segmental reiteration) in insect ventral ganglia has been found for aminergic, sensory, motor and interneurons, and to some extent also for peptidergic neurons (see [82]–[85]). In contrast, the organization of peptidergic neurons in the ventral ganglion of *Drosophila* larvae does not in general follow a strict segmental reiteration of peptidergic modules throughout the neuromeres. The only segmentally reiterated distribution throughout all ganglionic neuromeres or within a tagma was found for Pea-MIP neurons in neuromeres t1–a8 (Fig. 10), and the FMRFa-containing Tv neurons in the three thoracic neuromeres (Fig. 7). The Tv neurons innervate the thoracic neurohemal perisympathetic organs, which have a tagma-specific organization and peptidome and exclusively express FMRFa-like peptides across all insects studied (see [12], [13], [86]). The segmentally reiterated distribution of leucokinin and CCAP neurons within the abdominal neuromeres was restricted to a1–7 (Fig. 4, 9). Each leucokinin neuron sends a projection to the periphery with terminals on muscle M8 (the segmental border muscle, [67]) which is lacking in a8/9 (see [87]). This might functionally explain the lack of leucokinin neurons in a8/9. The distribution patterns of the other peptidergic neurons with somata in the abdominal neuromeres show even less serial homology. AST neurons are segment-specific and occur only in a1–2 (Fig. 2). Other peptidergic neuron types skip one or several neuromeres, e.g. corazonin neurons only occur in a1–a6 (Fig. 5). The lack of a strict segmentally reiterated pattern throughout the thoracic and abdominal neuromeres suggests that the restricted and differential distribution of peptidergic neurons reflects neuromere-specific functional connections. We are, however, not aware of other larval neuron types or circuits that match the observed peptidergic distribution patterns.

The last two abdominal neuromeres a8/9 have a unique pattern of peptidergic somata and projections (e.g. FMRFa, MIP or PDF neurons (Fig. 7, 10, 11)) and show the least serial homology to the more anterior neuromeres of the ventral ganglion. This finding also extends to descending processes. Descending axons may stop before or when reaching the border to a8 (HUG and DTK neurons (Fig. 3, 12)), form extensive varicose ramifications within the neuropil of a8 (AST, corazonin (Fig. 2, S3-C)) or branch extensively in the terminal plexus of a9 (FMRFa-, leucokinin-, MIP and PDF-neurons (Fig. 10, S4-B, S5-B, S6). Belonging to the tail region, the segments a8/9 differ from the homomeric segments a1-7 with respect to the organization of muscles and sensory neurons (see [27], [87]). Furthermore, several unique structures such as the spiracles or the anal pads belong to these terminal segments. Unlike other segmental nerves, the segmental nerve of a9 innervates the hindgut musculature [27]. The unique pattern of peptidergic neurons in a8/9 might thus, at least partially, reflect a segment-specific function related to e.g. control of spiracles or intestinal functions. For example, the PDF neurons innervate the hindgut [74], but their exact function is so far unknown. Similar segmental differences between a8 and the rest of the abdominal neuromeres have been found for neurons expressing biogenic amines [84].

### SYB.EGFP and RDL.HA as compartment markers in peptidergic neurons

The fusion construct *syb.egfp* has been developed as a presynaptic marker [88]. Since synaptobrevin (SYB) is an integral membrane protein of small synaptic vesicles and large peptide-containing vesicles alike, SYB.EGFP also labels peptide vesicles and hence peptide accumulation and release sites (varicosities), which typically do not spatially coincide with synapses. Concomitantly, we assume that purely dendritic compartments of peptidergic neurons do not contain vesicles and show no or only weak SYB.EGFP labeling. These assumptions are supported by results obtained for PDF neurons in the brain [6], and the Tv and Va neurons which innervate neurohemal organs (Fig. S1, Fig. S4-A3). Here indeed, SYB.EGFP was only found in the cell bodies (where the protein is made) and in the terminals in the neurohemal organs (where the peptidergic vesicles are stored and released). The axonal projections as well as the arborizations within the VNC were unlabeled. Nevertheless, when interpreting the SYB.EGFP distribution, it has to be kept in mind that SYB.EGFP might also label presynaptic sites if the peptidergic neurons contain colocalized classical neurotransmitters.

The haemagglutinin-tagged GABA_A_ receptor subunit RDL.HA has been shown to be a useful specific postsynaptic marker in motor neurons [89]. Since The GABA_A_ receptor subunit RDL is involved in mediating GABAergic postsynaptic currents [90], we tried whether ectopic RDL.HA expression indicates postsynaptic sites (dendrites) of peptidergic neurons also. The general expression level of RDL.HA was very weak, and we only obtained discernible labeling intensities with two different GAL4-drivers, *Ccap*- and c929-GAL4 (Fig. 13-D, Fig. S2-C). Nevertheless, the labeling was spatially very confined to neuron compartments that showed no varicosities or only weak SYB.EGFP fluorescence. This suggests that RDL.HA labeled postsynaptic sites in peptidergic neurons.

### Many peptidergic arborizations are concentrated around the ganglion midline and might define a dendritic neuropil compartment of peptidergic neurons

Arborizations around the median DM and VM tracts turned out to be a prominent feature of most characterized peptidergic neurons with somata in the ventral ganglion, including the AST, CCAP, corazonin, FMRFa, MIP and PDF neurons (Fig. 2, 4, 5, 7, 10, 11). In contrast to e.g. motor neurons, the prominent midline arborizations of peptidergic neurons were rather short, and did not occupy large areas in the more lateral neuropils between the median and lateral tracts. For the CCAP neurons, ectopically expressed RDL.HA localized exclusively to these median arborizations (Fig. S2-C). In contrast, SYB.EGFP as well as peptide-immunoreactivity was absent or relatively low in these arborizations (Fig. S2,4,6). Also in the general peptidergic c929-GAL4-line, SYB.EGFP expression was low in the median compared to lateral fascicles (Fig. S8). This might suggest that the median arborizations represent peptidergic dendrites. Descending processes of CCAP, EH, HUG and leucokinin neurons (originating from somata in the suboesophageal ganglion or the brain) all have putative release sites around the DM and VM tracts (Fig. 3, 4, 6, 9). Of the peptidergic neurons with cell bodies in the VNC, only those expressing corazonin were found to have varicosities indicative of release sites around the DM and VM tracts (Fig. 5).

Taken together, these findings suggest that the arborizations around the DM and VM tracts are mainly input compartments for peptidergic VNC neurons, and point to this midline region as a main site for synaptic inputs onto peptidergic neurons including the CCAP neurons. The different putative sites of in- and outputs to peptidergic neurons in the VNC are summarized in Table 3. Peptides released from varicosities of leucokinin, CCAP, HUG-, EH and corazonin neurites along the DM tract may modulate synaptic transmission around the DM tracts, or might represent direct input signals to peptidergic neurons. Also, the dorsal ap-let neurons with somata in the ventral ganglion expressing the peptide precursor Nplp1 appear to have their output sites along the DM tracts as indicated by strong peptide immunoreactivity [91]–[93]. Unlike any of the peptidergic neurons characterized here, the dorsal ap-let neurons seem to have extensive arborizations within the neuropil of each hemineuromere [91] which appear to contain no or only little peptide immunoreactive material [92], [93] and hence might represent dendritic regions. Also the leucokinin neurons with somata in the ventral ganglion do not send projections towards the midline (Fig. 9). Since leucokinin release is likely to occur at peripheral release sites on body wall muscles [67], it is possible that a synaptic input region is located along the VL tract, the only projection site of abdominal leucokinin neurons within the CNS neuropil.

**Table 3:** Assignment of putative main compartment identities as suggested by morphology, immunolabeling intensities and distribution of synaptic markers

Fas2 tract	peptide projection	putative compartment identity
DM/VM	AST-A	input
	CCAP	input (and non-peptidergic output?)
	corazonin	in?- and output
	MIP	in- and output
	EH	output
	c929	in- and output
DM	HUG-PK	output
	FMRFa	input (Tv neurons), output (descending neurons)
	leucokinin	output (descending neurons)
	PDF	input
VM	Dtk	n.d.
DL	AST-A	n.d.
	HUG-PK	output
	IFa	output
	c929	in- and output
VL	leucokinin	input?
	CCAP	output
	c929	in- and output
CI	AST-A	n.d.
	MIP	output
	Dtk	output
	c929	input
CL	corazonin	output
	MIP	output
	Dtk	output
	c929	in- and output
neuropil a8	corazonin	output
	c929	output
neuropil a9	FMRFa	n.d.
	leucokinin	output (descending neurons)
	c929	in- and output

### The neuropil of a9 is densely supplied by peptidergic neurites and might serve as in- and output site for peptidergic neurons

The last abdominal neuromere is only rudimentary [27] and does not display the typical Fas2 pattern seen in the rest of the ventral ganglion. Instead, the Fas2 tracts converge in the neuropil of a9 (also termed “terminal plexus” [24]), and seem to fill it completely (Fig. 1, Video S1, S2 and S3). Accordingly, the neuropil of a9 cannot be organized as the other ventral neuromeres. Still, a9 receives sensory afferents and also sends out motor efferents [27]. Based on the distribution pattern of ectopically expressed synaptic markers with the peptidergic c929-GAL4 driver and the differences in the distribution pattern of PDF immunoreactivity and *pdf*-GAL4-driven SYB.EGFP and CD8.GFP (Fig. 11, S6, S8), the terminal plexus seems to represent a prominent site for converging inputs to several peptidergic neurons. The presence of rather weak SYB.EGFP labeling, and varicose and strongly labeled endings of descending leucokinin neurites (Fig. S5) suggest that the terminal plexus at the same time also serves as an output neuropil for peptidergic neurons.

The termination of several descending peptidergic neurites just anterior to this terminal neuropil demonstrates that the existing peptidergic projections in the terminal plexus are neuromere-specific. The peptidergic innervation of the terminal plexus might thus reflect tail-specific input onto peptidergic neurons, or a functional role of the peptides in tail- or gut-related physiological processes (see above). A comparable terminal neuropil with dense peptidergic innervation appears to be present also in larvae of other fly species (based on leucokinin immunostaining [67]), but has not been described so far in adult flies or other insects with the possible exception of cockroaches. As in fly larvae, descending leucokinin neurites from the brain terminate and branch in a median neuropil in the last abdominal neuromere in the terminal abdominal ganglion of the Madeira cockroach *Leucophaea maderae* [68]. In the American cockroach, *Periplaneta americana*, the median dorso-caudal neuropil of the last abdominal neuromere shows dense pyrokinin- [94], perisulfakinin- [83], [95], as well as proctolinergic arborizations [96] which make both pre- and postsynaptic contacts to peptidergic and non-peptidergic neurons [97]. The corresponding posterior proctolinergic neurons of the cockroach send processes to the hindgut musculature [96]. Similar efferent proctolinergic neurons have been demonstrated in the ventral nerve cord of *Drosophila* and a blowfly species [98]–[100]. In *Periplaneta*, the region of the dorso-caudal neuropil contains a much higher diversity of neuropeptides than any other region in the ventral ganglion chain as shown by mass spectrometric profiling [101]. A similar situation might also apply to phasmids, mantids and isopods [101].

### Possible overlap of peptidergic and sensory projections

As outlined above, the area between the DM and VM tracts of each hemineuromere appears to be a main input region for peptidergic neurons of the VNC. This region does not appear to contain sensory projections, which instead are concentrated to the area between the ventrolateral and median tracts in each hemineuromere [24], [102]. Only the terminals of class I multidendritic neurons (vpda, ddaE and vbd neurons) and dbd neurons, multidendritic neurons with bipolar dendrites, are located laterally at the DM tracts [24]–[26], [102] and could thus potentially overlap with CCAP, corazonin, FMRFa or MIP arborizations. Class II–IV multidendritic neurons have been mapped to the ventral CNS [26]. This area is mostly outside the putative input area of peptidergic neurons between the DM and VM tract, only the TRP-neurites running along the VM tract might overlap. The more ventrally located median projections of further multidendritic neurons [24], [26] seem not to overlap with peptidergic projections. Neurites of CCAP, FMRFa, leucokinin and MIP along the VL tracts might, however, be close to the terminals of external sense organ and class II multidendritic neurons [26], [102].

The projection area of chordotonal organs has been mapped to ventral areas partially overlapping with the CI tracts, and medially extending nearly until the VM tract [24]. This rather large area contains processes of AST, MIP, and DTK neurons.

In insects, cholinergic multidendritic neurons function as touch, stretch- or proprioreceptors that respond to changes in body shape (see [103], [104]), but their function in *Drosophila* has not been determined so far. In Heteropterans, the stimulation of abdominal stretch receptors (multiterminal neurons) can trigger the release of diuretic hormone and the peptide hormone PTTH which initiates molting [105], [106]; the underlying neuronal pathways are unknown. The CCAP/MIP neurons that possibly overlap with class I multidendritic neurons play an essential role during ecdysis, wing inflation and tanning in *Drosophila* [42], [107] and express functional ACh receptors [8]. One might thus speculate that sensory information from multidendritic receptor neurons of the body wall may modulate the release activity of CCAP/MIP neurons in the context of ecdysis, wing inflation or tanning. Obviously, an overlap of multidendritic sensory neurons and peptidergic neurons has to be demonstrated properly, yet this example shows that the morphological mapping of peptidergic neurons can lead to testable functional hypotheses.

The varicose morphology suggests that the processes of AST, MIP and TRP neurons in proximity to projections of class II–IV multidendritic neurons, chordotonal and external sense organs are peptide release sites. It is thus possible that ASTs, MIPs and DTKs modulate sensory inputs onto central neurons. The functions of ASTs and MIPs in *Drosophila* are unknown, but DTKs have been shown to have a modulatory role in sensory processing during olfactory perception [81].

### Possible overlap of peptidergic and motor neuron projections

As in other insects, the dendritic compartments of motor neurons in the *Drosophila* larva are located in the dorsal neuropil of the ventral nerve cord. They occupy most of the neuropil area dorsal to the CI tracts, with exception of the area between the DM tracts [24]. Based on the presence of varicosities within the dorsal neuropil, the following peptides might be released within the motor input area: ASTs, FMRFa, IFa, hug-PK and DTKs (Fig. 2, 3, 7, 8). This morphological correlation raises the possibility that these peptides are implicated in the regulation of locomotor activity, which is testable. So far, there is only evidence that DTKs released within the CNS regulate the locomotor activity of adult flies [81].

### Possible overlap of peptidergic projections with interneurons

The relative scarcity of potential overlap between sensory and neuropeptide projections found in this study suggests that most peptidergic neurons of the ventral nerve cord do not receive monosynaptic input from sensory neurons. It is thus likely that peptidergic neurons in the ventral ganglion of *Drosophila* receive polyneuronal inputs of different sensory modalities via interneurons. This assumption is in line with the described association of neurites of the pCC interneuron as well as unspecified non-sensory neurons with the DM tract [24]. Nevertheless, too little information about the morphology and spatial distribution of interneuron projections exists to derive suggestions for neuronal connections with peptidergic neurons. The detailed studies on the organization of thoracic or abdominal ganglia in other insects also do not assign particular neurons or inputs to the region dorsal to the “ventral association center” [108], which corresponds to the region around the *Drosophila* DM tracts. Interestingly, the dense arborizations of the vasopressin-like immunoreactive (VPLI) neurons in the locust suboesophageal ganglion with synaptic contacts to a descending cholinergic interneuron are situated around the ganglion midline [109] between the dorsal and ventral median tract, which relate to the DM and VM tract of *Drosophila* [24]. The VPLI neurons represent, to our knowledge, the only case in which the cellular identity and effect of synaptic input onto a peptidergic insect neuron has been established. The spiking activity of these neurons is indirectly modulated by visual and mechanosensory input via the cholinergic interneuron [110].

The descending leucokinin, CCAP, corazonin and FMRFa-containing axons that run along the median side of the DM tracts (Fig. 4, 5, 7) suggest these peptides as candidate non-synaptic input factors for peptidergic neurons with arborizations around the DM tract.

The longitudinal axons of AST, FMRFa, MIP, and DTK neurons are associated with the CI tracts (Fig. 2, 7, 10, 12), and thus roughly located in an intermediate tier between the dorsal neuropil containing motor neuron dendrites, and the ventral neuropil containing sensory projections. Although it has been questioned whether it indeed reflects a functional organization [111], the middle tier has classically been assigned as “associative” neuropil containing interneurons [112], [113]. Peptides released at the CI tract region thus appear well suited to modulate interneurons, which in principle could provide an effective means to achieve wide ranging effects on different (interlinked) neuronal networks [114].

### Conclusions

Our mapping of the products of 12 peptide precursor genes suggests that peptidergic neurons in the *Drosophila* VNC typically have separated in- and output compartments in specific areas. Prominent input areas appear to be located around the DM/VM tracts, and in the terminal plexus of a9. Output areas within the VNC are less defined: for some descending and local peptidergic neurons release may be around DM/VM tracts, but others may release their peptides along the CI or lateral tracts.

The neuromere-specific distributions and morphological specializations of many peptidergic neurons indicate that subsets of peptidergic neurons of a given chemical identity may subserve differential and neuromere-specific functions. The prevailing lack of pronounced arborizations at putative output sites within the VNC and the distribution of varicosities and SYB.EGFP along the length of little branched descending fibers suggest that most of the investigated interneuronal peptides act via paracrine release and volume transmission [115]. However, our results cannot exclude more focal actions of peptides. The secretory neurons described here have peripheral release sites in the PSOs, the gut or at muscles.

Based on findings in vertebrates, molluscs and crustaceans (see [116]–[118]), neuropeptides in insect interneurons are commonly considered to be released as co-transmitters together with classical transmitters or biogenic amines (see [2]). The mapping of peptidergic neurons now allows to compare the distribution of peptides with that of classical neurotransmitters and biogenic amines in order to test the extent of co-transmission in the *Drosophila* ventral ganglion.

## Materials and Methods

### Fly stocks

Wild-type Oregon R (OrR), GAL4 driver fly strains and UAS reporter gene lines (see Table 1) were reared under a (12∶12) L:D cycle at 18°C or 25°C on standard cornmeal agar medium and yeast. GAL4 driver strain flies were crossed with UAS reporter gene fly strains to localize peptidergic neurons in the ventral nerve cord in the offspring.

### Immunostaining

CNS from third instar OrR or GAL4xUAS larvae were dissected in standard fly saline, fixed for 2 hours in 4% paraformaldehyde in 0.1M sodium phosphate buffered saline (PBS, pH 7.2), washed in PBS with 1% TritonX (PBT) and incubated for at least 24 h in PBT containing 10% normal goat serum in combination with rabbit anti-peptide or mouse mAbs (see Table 2). The mouse mAbs were obtained from the Developmental Studies Hybridoma Bank under the auspices of the NICHD and maintained by the University of Iowa. Preparations were then washed 5 times during a day with PBT and incubated for at least 24 h in PBT containing 10% normal goat serum with Cy2, Cy3- or Cy5-conjugated AffiniPure goat anti-mouse or goat anti-rabbit IgG (H+L; Jackson ImmunoResearch, Germany), used at a dilution of 1:2000. Preparations were subsequently washed for about 4 h, and then mounted in 80% glycerol diluted in phosphate buffer. To avoid compression of the preparations, small plastic spacers were placed between the slide and cover glass.

The nomenclature of the Fas2-IR tracts follows [24].

### Confocal microscopy

Confocal stacks were acquired in 0.4-0.5 µm steps along the z-axis by a confocal laser scanning microscope (Leica TCS SP2, Leica Microsystems Wetzlar, Germany, equipped with a 40× HCX PL APO oil immersion objective, n.a. = 1.25) with 8bit intensity resolution. To obtain 3D images for the panels, stacks were subjected to texture-based volume rendering (voltex) using Amira 3.1 software (Mercury Computer Systems GmbH, Berlin, Germany). Otherwise, maximum projections are shown generated by Leica Confocal Software 2.6 (Leica Microsystems Heidelberg GmbH, Germany).

### Mapping of immunostaining patterns

Obtained images were assembled using Adobe Photoshop 7.0. Schematic drawings of double labelings (anti-Fas2/anti-Peptide and anti-Fas2/GFP) in the ventral nerve cord were generated using Adobe Illustrator CS 11.0 software. The suboesophageal region of the ventral nerve cord was not considered in the schemes.

## Supporting Information

Figure S1 Detail of CAPA neurons. Dorsal view of a maximum projection of a preparation expressing VA-GAL4-driven SYB.EGFP. Unlike GFP, SYB.EGFP only labels the somata of the Va neurons (asterisks) and the proximal neurohemal part of the abdominal transverse nerves 1–3 (arrows). Scale bar = 50 µm. the abdominal transverse nerves 1–3 (arrows). Scale bar = 50 µm.
(0.12 MB TIF)

Figure S2 Details of CCAP neurons. A) Dorsal view of a maximum projection of a preparation expressing Ccap-GAL4-driven GFP (A1) immunostained against CCAP (A2) and merged image (A3). Median arborizations are only visible by GFP, whereas the immunostaining strongly labels median descending fibers in the suboesophageal ganglion and the thoracic and abdominal neuromeres. B) Dorsal view of a maximum projection of a preparation expressing Ccap-GAL4-driven SYB.EGFP. The distribution of SYB.EGFP is more similar to that of CCAP-IR (A2) than that of GFP (A1). C) Dorsal view of maximum projections of preparations expressing Ccap-GAL4-driven RDL.HA. Only the cell bodies and distinct staining around the midline are labeled. The linear structures are trachea detected by their autofluorescence. Due to the weak labeling intensity, the preparation had to be scanned with high sensitivity. D) Ccap-GAL4-driven GFP, lateral view of a voltex projection. The CCAP neurons in the suboesophageal (arrowhead) and thoracic neuromeres are ventrally located (asterisks), whereas the abdominal CCAP neurons are in a dorsal position. E) Ccap-GAL4-driven GFP, voltex projection of the neuromeres a7–9. The terminal plexus is marked by an arrow. Scale bars = 50 µm, E) 25 µm. RDL.HA immunolabeling and GFP and SYB.EGFP expression is shown in green, CCAP immunolabeling in red, Fas2 in magenta.
(4.29 MB TIF)

Figure S3 Details of corazonin neurons. A) Ventral view of corazonin-IR neurons in t3–a2. A1) Voltex projection, A2) Maximum projection. A3) Detail of the neurite projections of the corazonin neurons in a1, voltex projection. B) Corazonin-IR neurite projections in a4–a6, maximum projections. B1–2) Dorsal view. B3) Ventral view of the neurite projections between the VM tracts in the segments a4–5. C1–2) Ventral view of a voltex projection of the abdominal segments a6–a8 showing the posteriormost pair of corazonin neurons in a6 and the dense pronounced varicosities in a7 and a8. C3) Posterior view of a cross section of a maximal projection in segment a7. Scale bars = 100 µm, B3) 25 µm. Immunostaining is shown in green, Fas2 in magenta.
(3.23 MB TIF)

Figure S4 Details of FMRFa neurons. A) fmrf-GAL4-driven expression of marker molecules in the Tv neurons. A1) GFP-expression, dorso-lateral view. A2) GFP-expression, lateral view. A3) SYB.EGFP-expression, dorsal view. The pronounced median arborizations in A1-2 (arrows) visualized by GFP are devoid of SYB.EGFP fluorescence in A3. SYB.EGFP is however accumulated in the thoracic PSOs (arrowheads) and the Tv neuron somata (asterisks). B) FMRFa-IR in the posterior abdominal neuromeres, dorsal view. Terminal plexus is marked by an arrow. Scale bars = A) 10 µm, B) 25 µm. Immunostaining or marker protein expression is shown in green, Fas2 in magenta.
(1.41 MB TIF)

Figure S5 Details of leucokinin neurons. A) Ventral view of a voltex projection of leucokinin neurons in a hemineuromere of a1–a3. B) Dorsal view of a voltex projection of leucokinin neurons in a5–a9 showing dense arborizations in the terminal plexus (arrow). C1–C3) Dorsal, posterior and lateral view of the dense arborizations in the terminal plexus. Scale bars = A–B) 30 µm, C) 10 µm. Immunostaining is shown in green, Fas2 in magenta.
(2.45 MB TIF)

Figure S6 Details of the morphology of PDF expressing neurons. PDF-neurons in a8–9, voltex projections. A) Lateral view. B) Anteriolateral view. C) Dorsolateral view. The arborizations in the terminal plexus (arrow) are only labeled by CD8.GFP (A), but not by immunostaining (C1) or SYB.EGFP (C2). Scale bars = 20 µm. Immunostaining or marker protein expression is shown in green, Fas2 in magenta.
(1.10 MB TIF)

Figure S7 Details of the morphology of TRP neurons. Voltex projections of TRP-IR varicosities in different neuromeres. A) Dorsal view of a3–a4. B) Posterior view of transverse section at the height of t3. C) Dorsal view of a pair of TRP-IR neurons (asterisks) in t2. D) Dorsal view of a3. Scale bars = A) 20 µm, B–D) 30 µm. Immunostaining is shown in green, Fas2 in magenta.
(2.78 MB TIF)

Figure S8 Distribution of c929-GAL4 driven expression of SYB.EGFP and CD8.GFP. Dorsal view of maximum projections of the VNC in gray scale (GS) or false color coding (FC). In FC, low staining intensity is coded by blue, high staining intensity by red. Each row represents a separate preparation. Strongest accumulation of SYB.EGFP is visible in the thoracic PSOs (arrows) and descending lateral and intermediate fascicles, whereas the terminal plexus (arrowhead) only shows relatively little fluorescence. In contrast, CD8.GFP fluorescence is most intense in the terminal plexus and in median and lateral fascicles, and missing in the thoracic PSOs. Scale bars = 100 µm.
(4.71 MB TIF)

Video S1 3D reconstruction of the Fas2 landmarks. Movie of a full reconstruction of the Fas2-positive tracts in the ventral nerve cord of a L3 larva.
(7.81 MB AVI)

Video S2 Moving layer through Fas2 landmarks, dorsoventral axis. Movie of the Fas2-positive tracts in the ventral nerve cord of a L3 larva, from ventral to dorsal.
(10.02 MB AVI)

Video S3 Moving layer through Fas2 landmarks, anterior-posterior axis. Movie of the Fas2-positive tracts in the ventral nerve cord of a L3 larva.
(9.27 MB AVI)
