# Supplementary figures and images for: Neuroarchitecture of Peptidergic Systems in the Larval Ventral Ganglion of Drosophila melanogaster
Source: PLoS One. 2007 Aug 1;2(8):e695. doi: 10.1371/journal.pone.0000695 (PMC1933254; doi:10.1371/journal.pone.0000695)

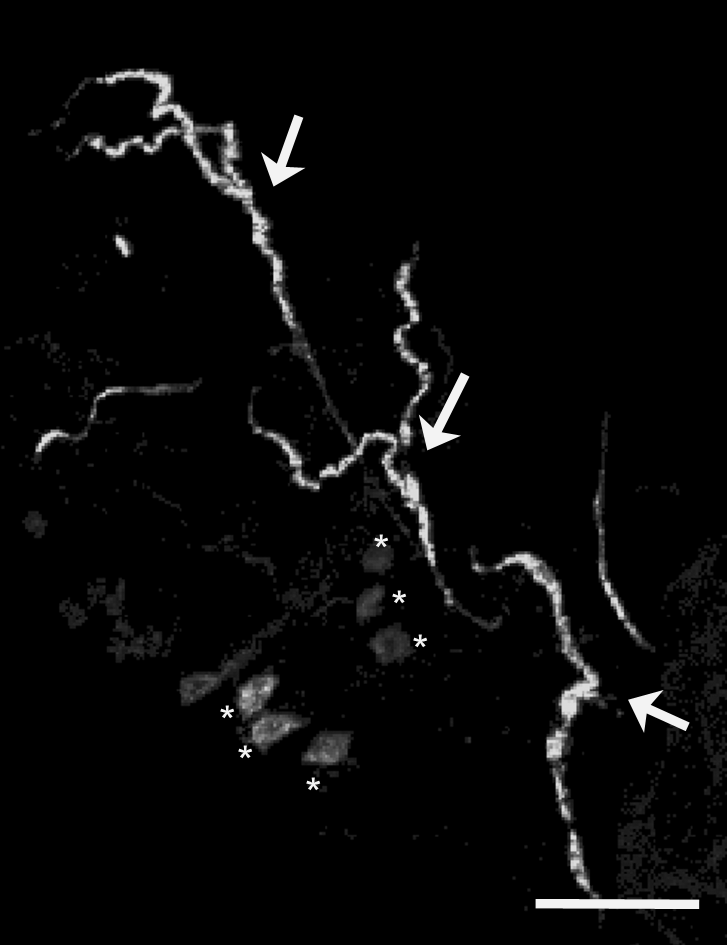

Supplement: Figure S1 — Detail of CAPA neurons. Dorsal view of a maximum projection of a preparation expressing VA-GAL4-driven SYB.EGFP. Unlike GFP, SYB.EGFP only labels the somata of the Va neurons (asterisks) and the proximal neurohemal part of the abdominal transverse nerves 1–3 (arrows). Scale bar = 50 µm. the abdominal transverse nerves 1–3 (arrows). Scale bar = 50 µm. (0.12 MB TIF) [file pone.0000695.s001.tif]

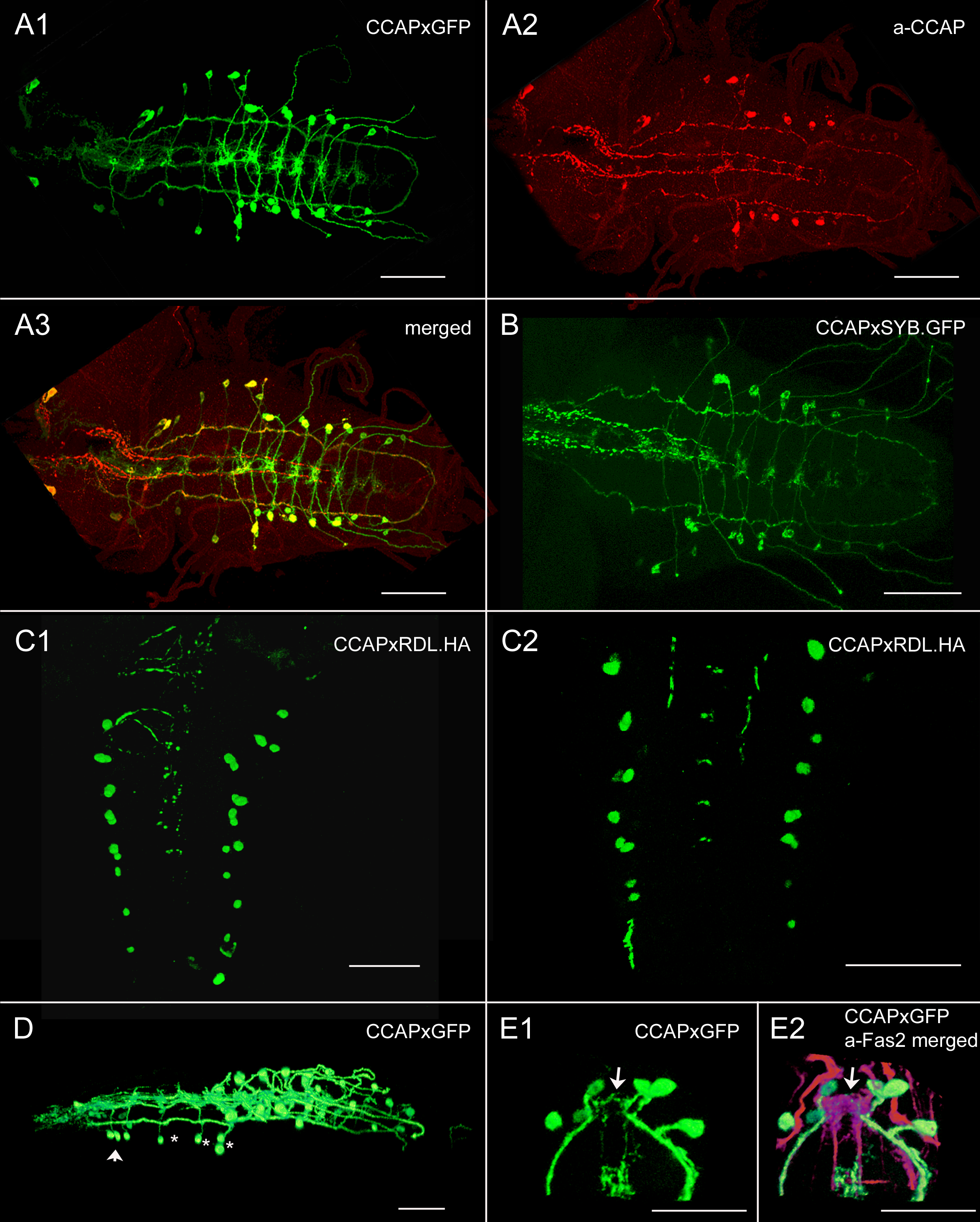

Supplement: Figure S2 — Details of CCAP neurons. A) Dorsal view of a maximum projection of a preparation expressing Ccap-GAL4-driven GFP (A1) immunostained against CCAP (A2) and merged image (A3). Median arborizations are only visible by GFP, whereas the immunostaining strongly labels median descending fibers in the suboesophageal ganglion and the thoracic and abdominal neuromeres. B) Dorsal view of a maximum projection of a preparation expressing Ccap-GAL4-driven SYB.EGFP. The distribution of SYB.EGFP is more similar to that of CCAP-IR (A2) than that of GFP (A1). C) Dorsal view of maximum projections of preparations expressing Ccap-GAL4-driven RDL.HA. Only the cell bodies and distinct staining around the midline are labeled. The linear structures are trachea detected by their autofluorescence. Due to the weak labeling intensity, the preparation had to be scanned with high sensitivity. D) Ccap-GAL4-driven GFP, lateral view of a voltex projection. The CCAP neurons in the suboesophageal (arrowhead) and thoracic neuromeres are ventrally located (asterisks), whereas the abdominal CCAP neurons are in a dorsal position. E) Ccap-GAL4-driven GFP, voltex projection of the neuromeres a7–9. The terminal plexus is marked by an arrow. Scale bars = 50 µm, E) 25 µm. RDL.HA immunolabeling and GFP and SYB.EGFP expression is shown in green, CCAP immunolabeling in red, Fas2 in magenta. (4.29 MB TIF) [file pone.0000695.s002.tif]

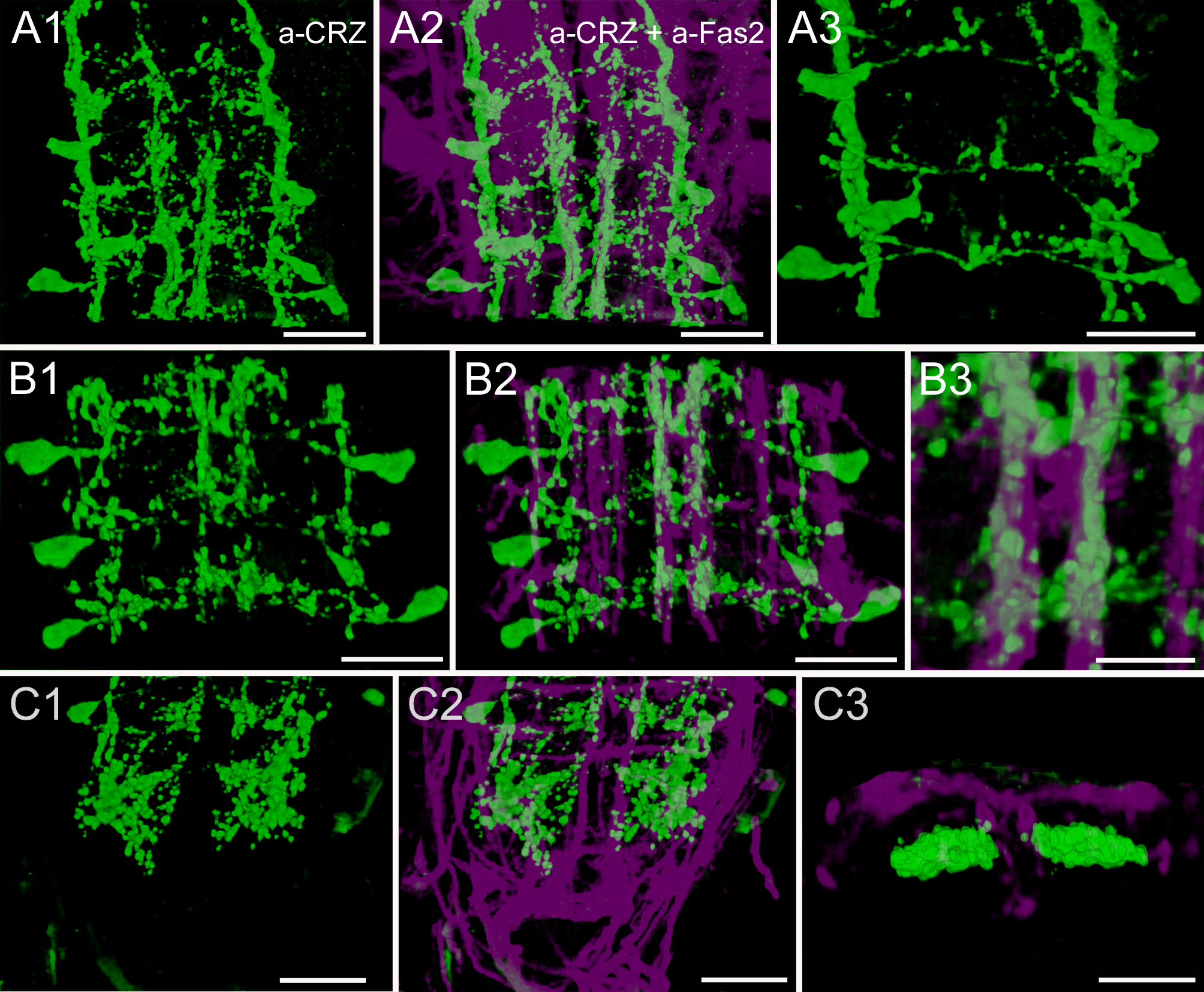

Supplement: Figure S3 — Details of corazonin neurons. A) Ventral view of corazonin-IR neurons in t3–a2. A1) Voltex projection, A2) Maximum projection. A3) Detail of the neurite projections of the corazonin neurons in a1, voltex projection. B) Corazonin-IR neurite projections in a4–a6, maximum projections. B1–2) Dorsal view. B3) Ventral view of the neurite projections between the VM tracts in the segments a4–5. C1–2) Ventral view of a voltex projection of the abdominal segments a6–a8 showing the posteriormost pair of corazonin neurons in a6 and the dense pronounced varicosities in a7 and a8. C3) Posterior view of a cross section of a maximal projection in segment a7. Scale bars = 100 µm, B3) 25 µm. Immunostaining is shown in green, Fas2 in magenta. (3.23 MB TIF) [file pone.0000695.s003.tif]

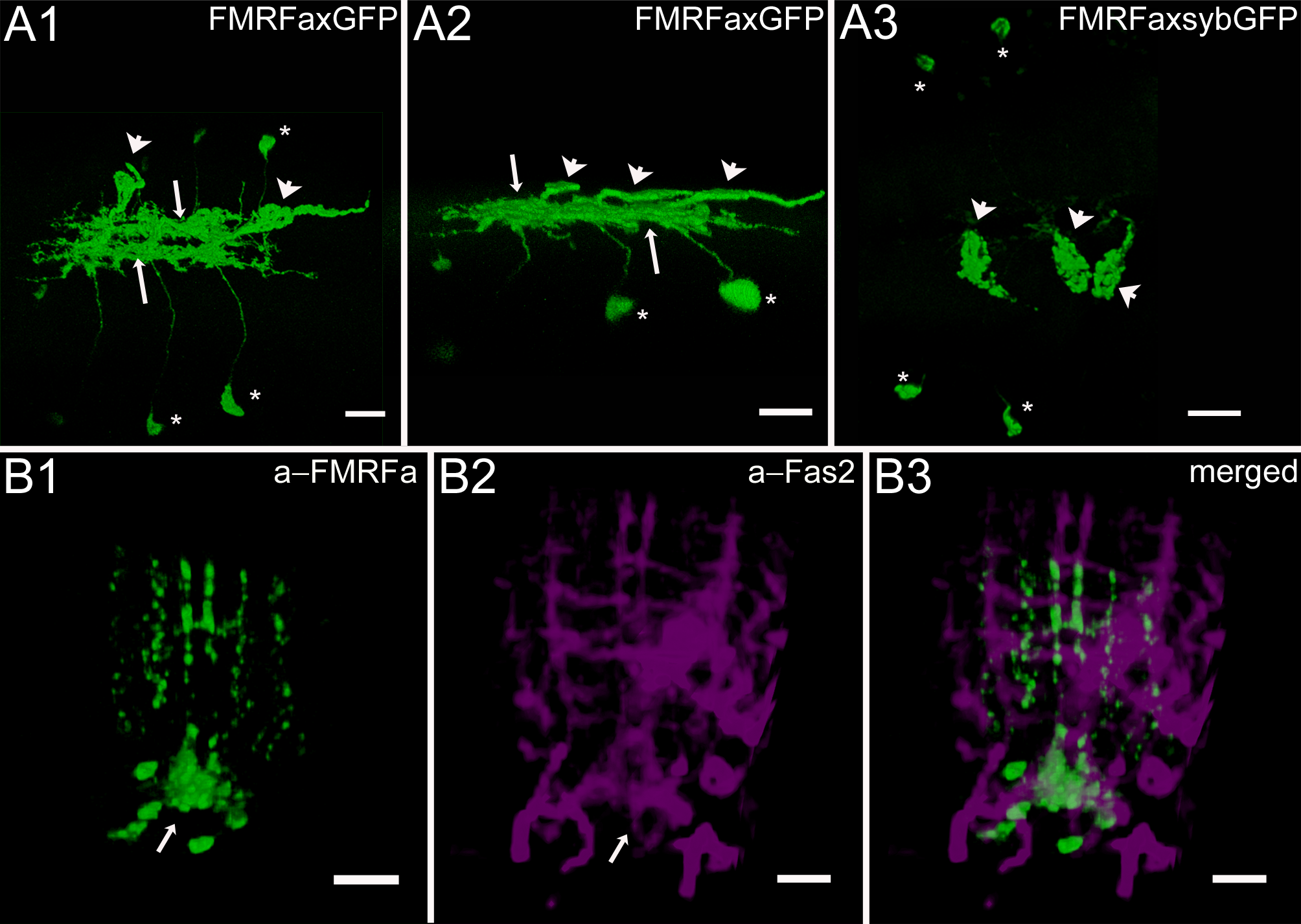

Supplement: Figure S4 — Details of FMRFa neurons. A) fmrf-GAL4-driven expression of marker molecules in the Tv neurons. A1) GFP-expression, dorso-lateral view. A2) GFP-expression, lateral view. A3) SYB.EGFP-expression, dorsal view. The pronounced median arborizations in A1-2 (arrows) visualized by GFP are devoid of SYB.EGFP fluorescence in A3. SYB.EGFP is however accumulated in the thoracic PSOs (arrowheads) and the Tv neuron somata (asterisks). B) FMRFa-IR in the posterior abdominal neuromeres, dorsal view. Terminal plexus is marked by an arrow. Scale bars = A) 10 µm, B) 25 µm. Immunostaining or marker protein expression is shown in green, Fas2 in magenta. (1.41 MB TIF) [file pone.0000695.s004.tif]

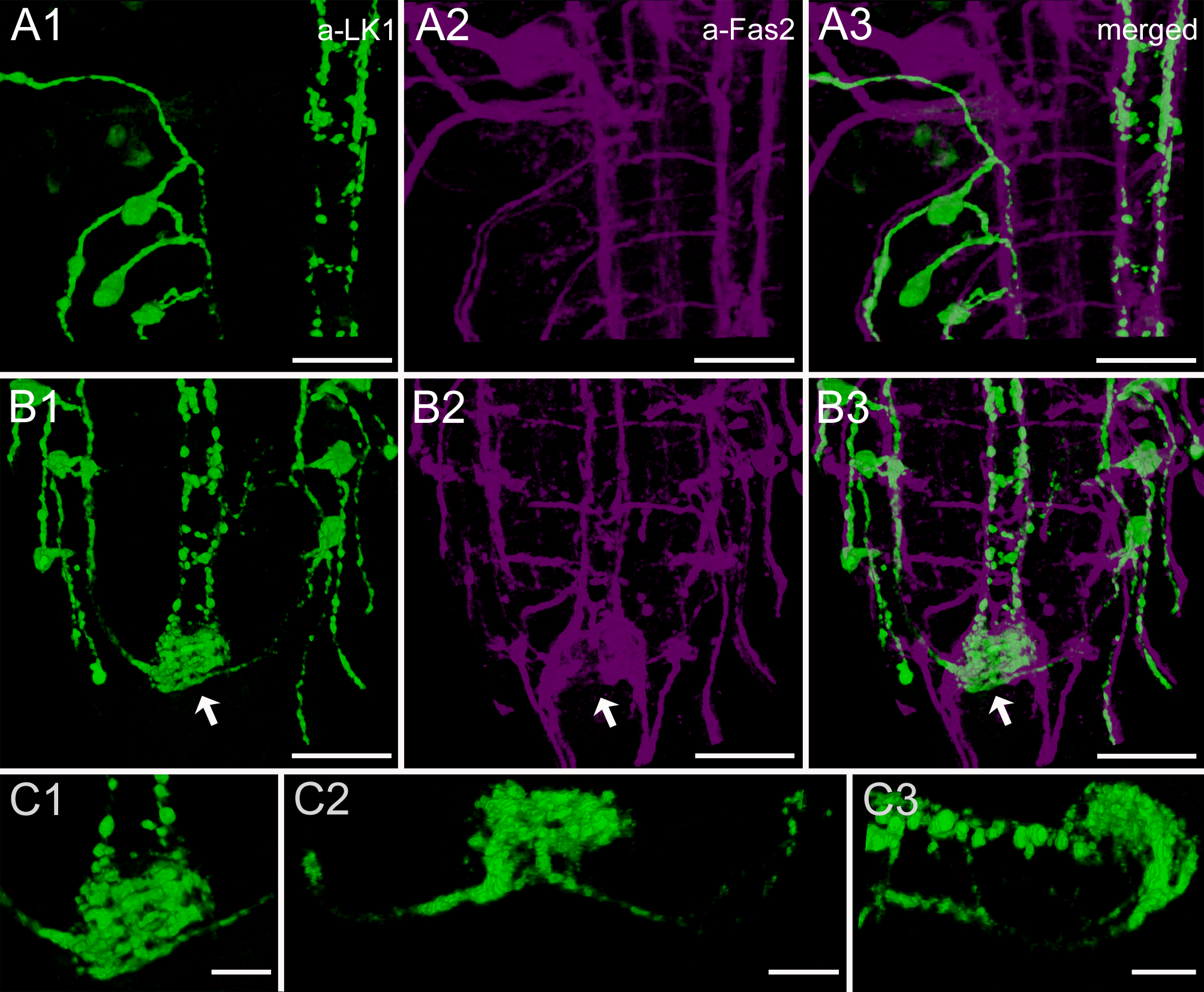

Supplement: Figure S5 — Details of leucokinin neurons. A) Ventral view of a voltex projection of leucokinin neurons in a hemineuromere of a1–a3. B) Dorsal view of a voltex projection of leucokinin neurons in a5–a9 showing dense arborizations in the terminal plexus (arrow). C1–C3) Dorsal, posterior and lateral view of the dense arborizations in the terminal plexus. Scale bars = A–B) 30 µm, C) 10 µm. Immunostaining is shown in green, Fas2 in magenta. (2.45 MB TIF) [file pone.0000695.s005.tif]

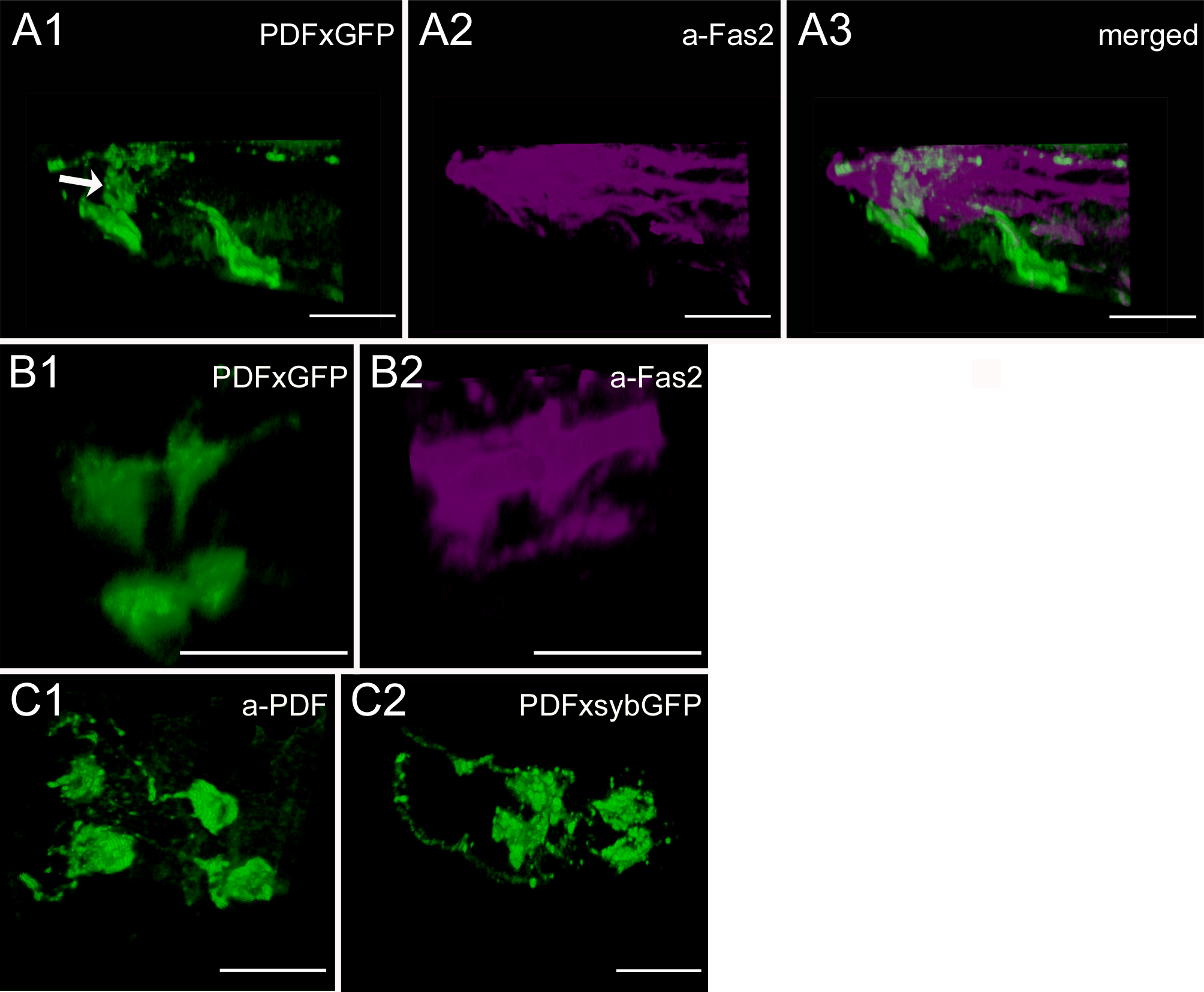

Supplement: Figure S6 — Details of the morphology of PDF expressing neurons. PDF-neurons in a8–9, voltex projections. A) Lateral view. B) Anteriolateral view. C) Dorsolateral view. The arborizations in the terminal plexus (arrow) are only labeled by CD8.GFP (A), but not by immunostaining (C1) or SYB.EGFP (C2). Scale bars = 20 µm. Immunostaining or marker protein expression is shown in green, Fas2 in magenta. (1.10 MB TIF) [file pone.0000695.s006.tif]

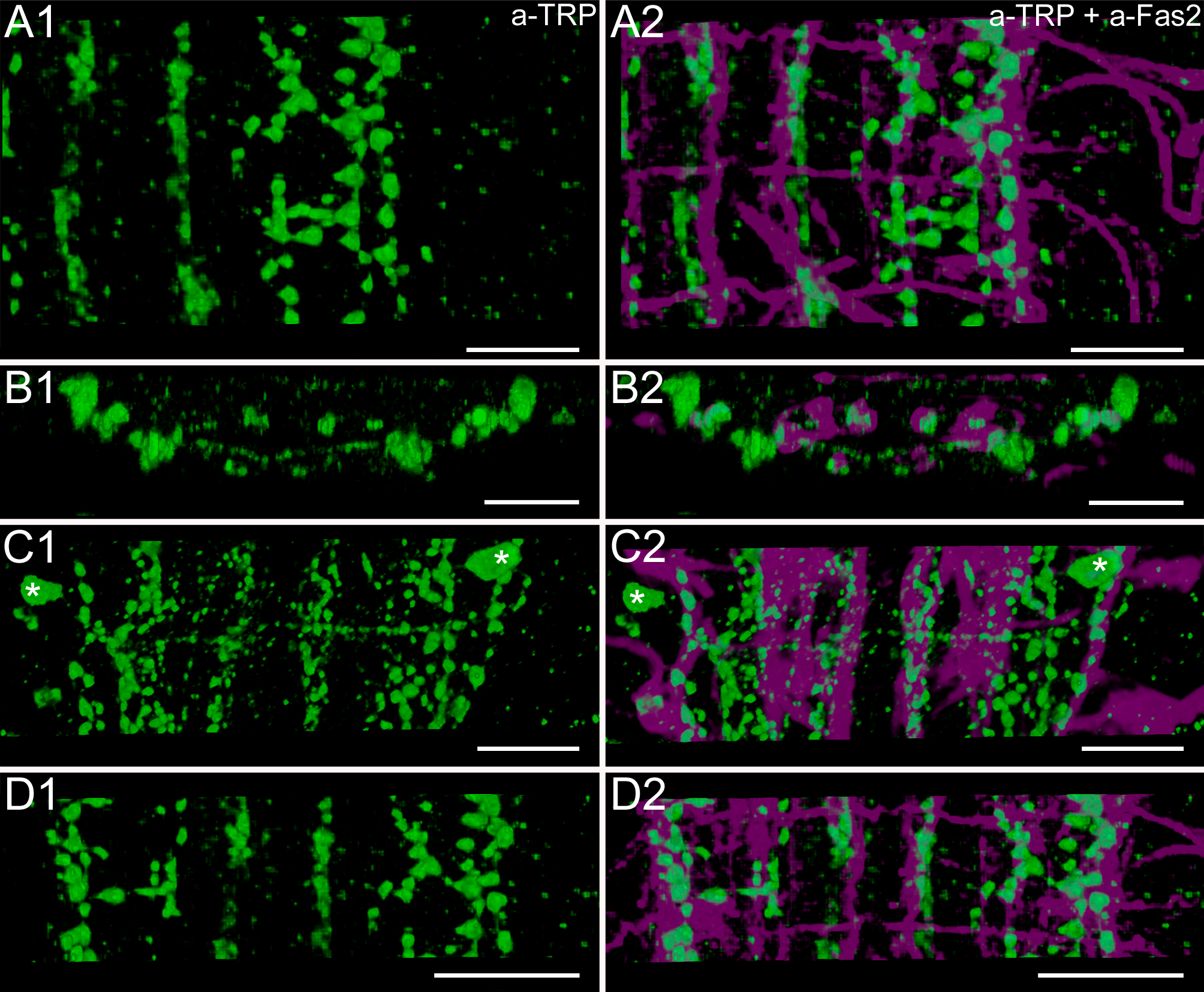

Supplement: Figure S7 — Details of the morphology of TRP neurons. Voltex projections of TRP-IR varicosities in different neuromeres. A) Dorsal view of a3–a4. B) Posterior view of transverse section at the height of t3. C) Dorsal view of a pair of TRP-IR neurons (asterisks) in t2. D) Dorsal view of a3. Scale bars = A) 20 µm, B–D) 30 µm. Immunostaining is shown in green, Fas2 in magenta. (2.78 MB TIF) [file pone.0000695.s007.tif]

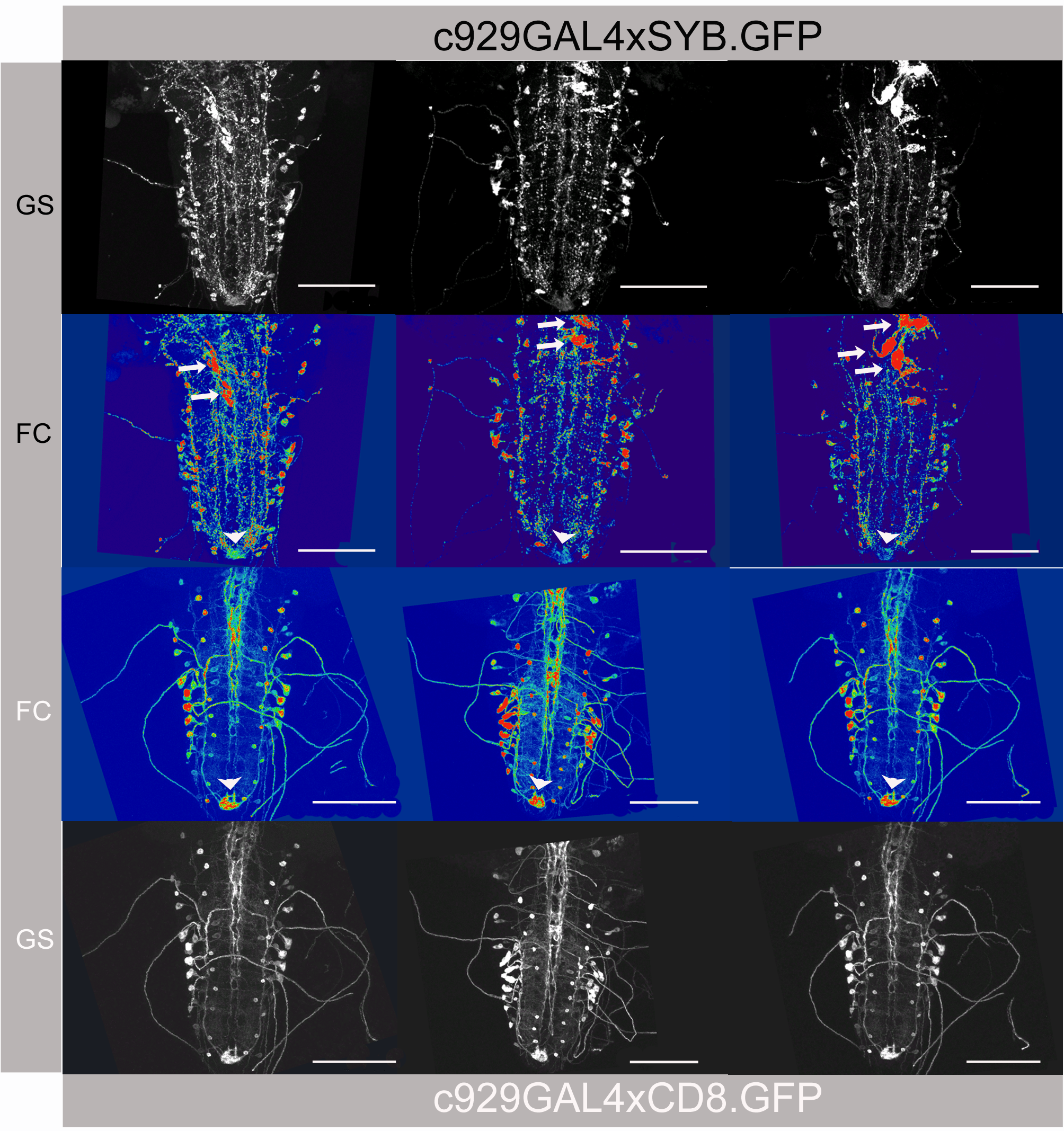

Supplement: Figure S8 — Distribution of c929-GAL4 driven expression of SYB.EGFP and CD8.GFP. Dorsal view of maximum projections of the VNC in gray scale (GS) or false color coding (FC). In FC, low staining intensity is coded by blue, high staining intensity by red. Each row represents a separate preparation. Strongest accumulation of SYB.EGFP is visible in the thoracic PSOs (arrows) and descending lateral and intermediate fascicles, whereas the terminal plexus (arrowhead) only shows relatively little fluorescence. In contrast, CD8.GFP fluorescence is most intense in the terminal plexus and in median and lateral fascicles, and missing in the thoracic PSOs. Scale bars = 100 µm. (4.71 MB TIF) [file pone.0000695.s008.tif]
